# A tRNA modification with aminovaleramide facilitates AUA decoding in protein synthesis

**DOI:** 10.1038/s41589-024-01726-x

**Published:** 2024-09-19

**Authors:** Kenjyo Miyauchi, Satoshi Kimura, Naho Akiyama, Kazuki Inoue, Kensuke Ishiguro, Thien-Son Vu, Veerasak Srisuknimit, Kenta Koyama, Gosuke Hayashi, Akiko Soma, Asuteka Nagao, Mikako Shirouzu, Akimitsu Okamoto, Matthew K. Waldor, Tsutomu Suzuki

**Affiliations:** 1 Department of Chemistry and Biotechnology, Graduate School of Engineering, The University of Tokyo, Tokyo, Japan; 2 Division of Infectious Diseases, Brigham and Women’s Hospital, Boston, MA USA; 3 Department of Microbiology, Harvard Medical School, Boston, MA USA; 4 Howard Hughes Medical Institute, Boston, MA USA; 5 Laboratory for Protein Functional and Structural Biology, RIKEN Center for Biosystems Dynamics Research, Yokohama, Japan; 6 Department of Biomolecular Engineering, Nagoya University, Nagoya, Japan; 7 Graduate School of Horticulture, Chiba University, Matsudo, Japan; 8 Present Address: Department of Microbiology and Immunology, College of Veterinary Medicine, Cornell University, Ithaca, NY USA; 9 Present Address: Department of Biochemistry, Chulalongkorn University, Bangkok, Thailand

**Keywords:** RNA modification, Plant signalling, Chemical modification

## Abstract

Modified tRNA anticodons are critical for proper mRNA translation during protein synthesis. It is generally thought that almost all bacterial tRNAs^Ile^ use a modified cytidine—lysidine (L)—at the first position (34) of the anticodon to decipher the AUA codon as isoleucine (Ile). Here we report that tRNAs^Ile^ from plant organelles and a subset of bacteria contain a new cytidine derivative, designated 2-aminovaleramididine (ava^2^C). Like L34, ava^2^C34 governs both Ile-charging ability and AUA decoding. Cryo-electron microscopy structural analyses revealed molecular details of codon recognition by ava^2^C34 with a specific interaction between its terminal amide group and an mRNA residue 3′-adjacent to the AUA codon. These findings reveal the evolutionary variation of an essential tRNA modification and demonstrate the molecular basis of AUA decoding mediated by a unique tRNA modification.

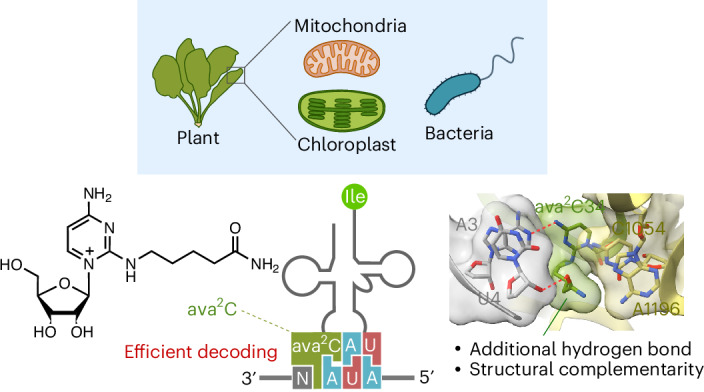

## Abstract (web summary)

A tRNA modification, named 2-aminovaleramididine (ava^2^C), has been discovered in plant organelles and bacteria. Structural analysis shows specific codon recognition by this modification, highlighting the evolutionary diversity of the essential tRNA modifications.

## Main

Approximately 150 RNA modifications have been found in various RNA molecules from all domains of life^1^. Among RNA species, tRNA contains the widest variety and largest number of RNA modifications^1,2^. tRNA modifications have pivotal roles in the maturation, stability and function of tRNAs. Modifications in the anticodon loop directly fine-tune codon recognition and amino acid charging abilities^2,3^. By contrast, modifications in the tRNA body region generally stabilize tRNA tertiary structure, preventing their cellular degradation^4,5^. The physiological importance of tRNA modifications is demonstrated by the finding that various human diseases are caused by their deficiency and/or misregulation^2,6–8^. The repertoire of tRNA modification differs among organisms. Although tRNA modification has been well characterized in some model organisms, such as *Escherichia coli*, yeast and humans, their profile and function in nonmodel organisms remain largely elusive. Recent studies of tRNAs from nonmodel organisms, including pathogenic bacteria, archaea and organelles, have uncovered new tRNA modifications and their regulation and physiological roles^9–11^.

A wide variety of tRNA modifications are clustered in the anticodon loop, particularly at the first letter of the anticodon (position 34)^2^. These so-called ‘wobble’ modifications are crucial for maintaining accurate and efficient codon recognition at the A-site of the ribosome during translation. Base pairing between the first position of the tRNA anticodon and the third position of the mRNA codon does not always obey Watson–Crick base pairing but can form noncanonical base pairing geometry. Wobble modifications are also frequently used as tRNA identity elements for charging cognate amino acids by aminoacyl-tRNA synthetases^2^ and can be essential for cell viability^12–14^.

**Extended Data Fig. 1:**
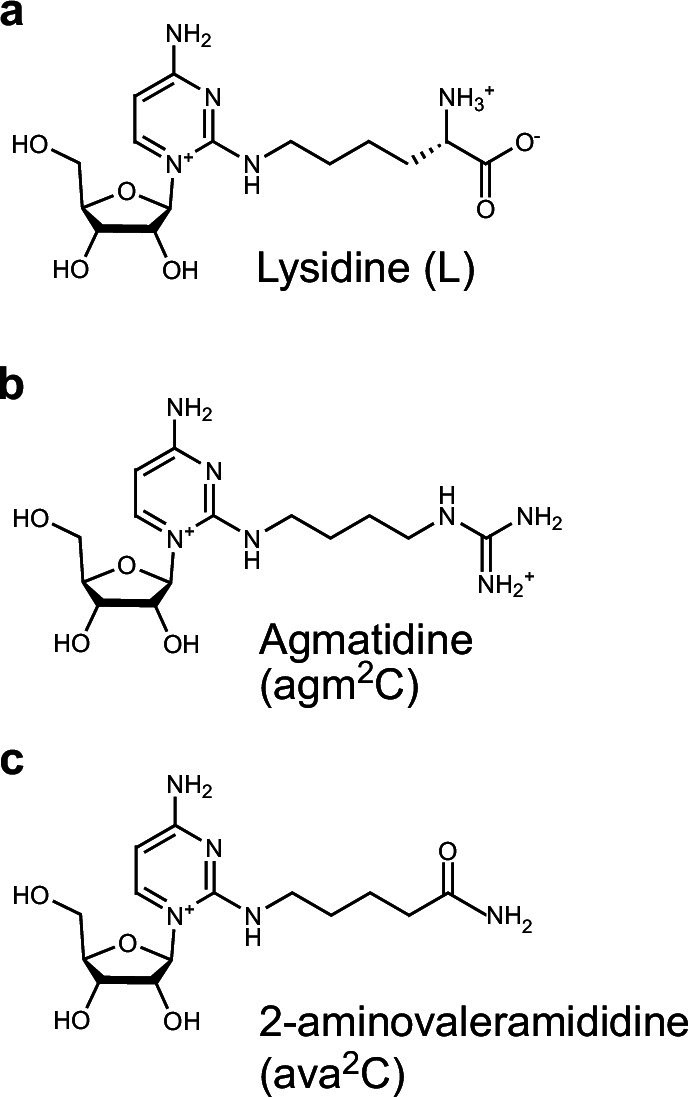
Chemical structures of tRNA modifications required for AUA codon decoding. The chemical structures of L (**a**), agm^2^C (**b**) and ava^2^C (**c**) are shown as their enamine isoforms.

In general, each of the purine (R)-ending two-codon sets, denoted as NNR (NNA and NNG), specifies a single amino acid because NNR codons are usually decoded by a tRNA having modified uridine at position 34 together with another isoacceptor with C34 (ref. ^15^). AUA and AUG (AUR) codons are exceptions and specify isoleucine (Ile) and methionine (Met), respectively, and are separately decoded by tRNAs for Ile and Met^16^. Modified uridine at position 34 cannot distinguish A and G, making it difficult for organisms to differentially decode AUA as Ile and AUG as Met. Each domain of organisms has evolved sophisticated systems for AUR decoding mediated by diverse tRNA modifications^17^. In eukaryotes, tRNA^Ile^ with inosine (I) at position 34 likely reads the AUA, AUU and AUC codons, whereas tRNA^Met^ with the CAU anticodon only recognizes the AUG codon. Presumably, the AUA codon is redundantly read by another isoacceptor with pseudouridine (*Ψ*) at position 34 (ref. ^17^). In contrast, bacteria and archaea adopt modified cytidines in tRNA^Ile2^, which is responsible for AUA decoding. Bacteria have tRNA^Ile2^ bearing lysidine (2-lysyl cytidine, L; Extended Data Fig. 1a) at position 34 (ref. ^18^). L is a modified cytidine conjugated with lysine at the C2 of cytosine. The precursor tRNA^Ile2^ with the CAU anticodon is methionylated and reads the AUG codon^19^ and thus behaves like tRNA^Met^. Once L34 is introduced by the enzyme TilS, the mature tRNA^Ile2^ is isoleucylated and reads the AUA codon^14^. Also, L34 prevents misdecoding of the AUG codon. Thus, both the amino acid and codon specificities of tRNA^Ile2^ rely on a single L34 modification^14,19^. The necessity of L34 for AUA decoding was demonstrated biochemically^19^ and genetically because *tilS* is an essential gene^14,20^. TilS homologs are widely distributed in bacteria, suggesting L is highly conserved across bacterial species. In archaea, agmatidine, a modified cytidine (2-agmatinylcytidine (agm^2^C); Extended Data Fig. 1b) occurs at position 34 of tRNA^Ile2^ (refs. ^21,22^) and functions similarly to L34 for Ile charging and AUA decoding. Despite the similarity in the chemical structures of L and agm^2^C, distinct classes of enzymes and catalytic mechanisms account for their biosynthesis^17,23^. L34 is synthesized by TilS, an N-type ATP pyrophosphatase family protein^14,24,25^, whereas agm^2^C34 is synthesized by TiaS, which has a kinase domain with dual specificity for RNA and protein^23,26^. These facts highlight the apparent convergent evolution of AUA decoding by tRNA^Ile2^ with modified cytidines between bacteria and archaea^17^.

Recently, we and another group reported the cryo-electron microscopy (EM) structures of tRNAs^Ile^ deciphering the AUA codon on the ribosome and revealed the molecular basis of AUA decoding mediated by L34 (refs. ^27,28^) and agm^2^C34 (ref. ^27^) on the ribosome. Both cytidine modifications base pair with the third adenine of the AUA codon via a unique C–A geometry. The long side chains extend toward the 3′ direction of the AUA codon to fit into the cleft formed by rRNA residues and the mRNA strand, and the polar termini form a hydrogen bond with 2′-OH of the mRNA residue 3′-adjacent to the AUA codon. Biochemical studies revealed that AUA decoding is facilitated by this additional hydrogen bond between the polar termini of the modified cytidines and 2′-OH of the mRNA residue. These analyses suggested that bacteria and archaea adopt largely common but partly specific mechanisms to stabilize codon–anticodon pairing with distinct cytidine modifications. The variations of the terminal chemical moieties likely fine-tune decoding.

In most animal mitochondria, the AUA codon specifies Met, not Ile. In contrast, it specifies Ile in plant organelles, chloroplasts and mitochondria, indicating the presence of modified cytidine in plant organellar tRNAs^Ile^. The codon-specific tRNAs^Ile^ isolated from spinach chloroplasts^29^ and potato mitochondria^30^ have modified cytidines at position 34, but the chemical structures of these modifications were unknown. Here we report a new cytidine derivative, named 2-aminovaleramididine (ava^2^C; Extended Data Fig. 1c), found in tRNA^Ile^ from plant organelles and a subset of bacteria. ava^2^C is a cytidine derivative conjugated with 5-aminovaleramide (5-AVA, also known as 5-aminopentanamide) at position C2. *tilS* is involved in ava^2^C formation in *Vibrio cholerae*, suggesting that ava^2^C is synthesized by additional modification of L. Additionally, like L, ava^2^C34 in tRNA^Ile2^ is required for isoleucylation by isoleucyl-tRNA synthetase (IleRS) and AUA decoding on the ribosome. Cryo-EM structural analyses of the 70S ribosome complexed with tRNA^Ile2^-bearing ava^2^C34 suggest the molecular mechanism by which ava^2^C34 facilitates AUA codon recognition at the A-site of the ribosome.

## Results

### N^341^ is observed in plant and bacterial tRNA^Ile2^

**Fig. 1:**
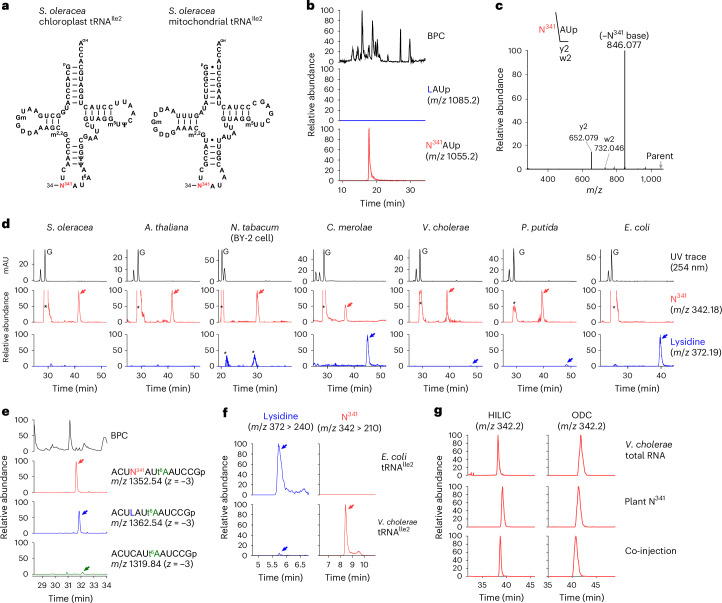
N^341^ is a modified cytidine at position 34 of tRNA^Ile2^ in plant organelles and bacteria. **a**, Secondary structures of tRNA^Ile2^ from spinach chloroplasts (left) and mitochondria (right). The modifications other than N^341^ in chloroplast tRNA^Ile2^ were reported previously^29^ and confirmed by LC–MS analyses (Extended Data Fig. 2a,b and Supplementary Table 1a,b). For mitochondrial tRNA^Ile2^, the modifications other than *Ψ* were mapped by LC–MS analysis (Extended Data Fig. 2c and Supplementary Table 1c). **b**, LC–MS analysis of RNase A digests of spinach chloroplast tRNA^Ile2^. The BPC (top) and XICs for L-containing (middle) and N^341^-containing (bottom) fragments are shown. **c**, CID spectrum of the N^341^-containing fragment of spinach chloroplast tRNA^Ile2^ digested by RNase A. The product ions are indicated on the CID spectrum and assigned to the corresponding sequence. **d**, Nucleoside analyses of total RNA from various plants and bacterial species. The UV trace (top) and mass chromatograms detecting proton adducts of N^341^(middle) and L (bottom) are shown. The red arrows indicate the N^341^ peaks, and the blue arrows indicate the lysidine peaks. **e**, LC–MS analysis of RNase T_1_ digests of *C. merolae* chloroplast tRNA^Ile2^. The BPC (top) and XICs for N^341^-containing (second), L-containing (third) and C-containing (bottom) fragments are shown. **f**. Nucleoside analysis of *E. coli* and *V. cholerae* tRNA^Ile2^. MRM chromatograms detecting L (*m*/*z* 372 > 240) and N^341^ (*m*/*z* 342 > 210) are shown. **g**, LC–MS co-injection analyses of bacterial and plant N^341^. N^341^ from *V. cholerae* (top) and plants (middle), and a co-injected sample (bottom) were analyzed by HILIC (left) and ODS columns (right). XICs detecting N^341^ (*m*/*z* 342.2) are shown. BPC, base peak chromatogram; XICs, extracted ion chromatograms.

**Extended Data Fig. 2:**
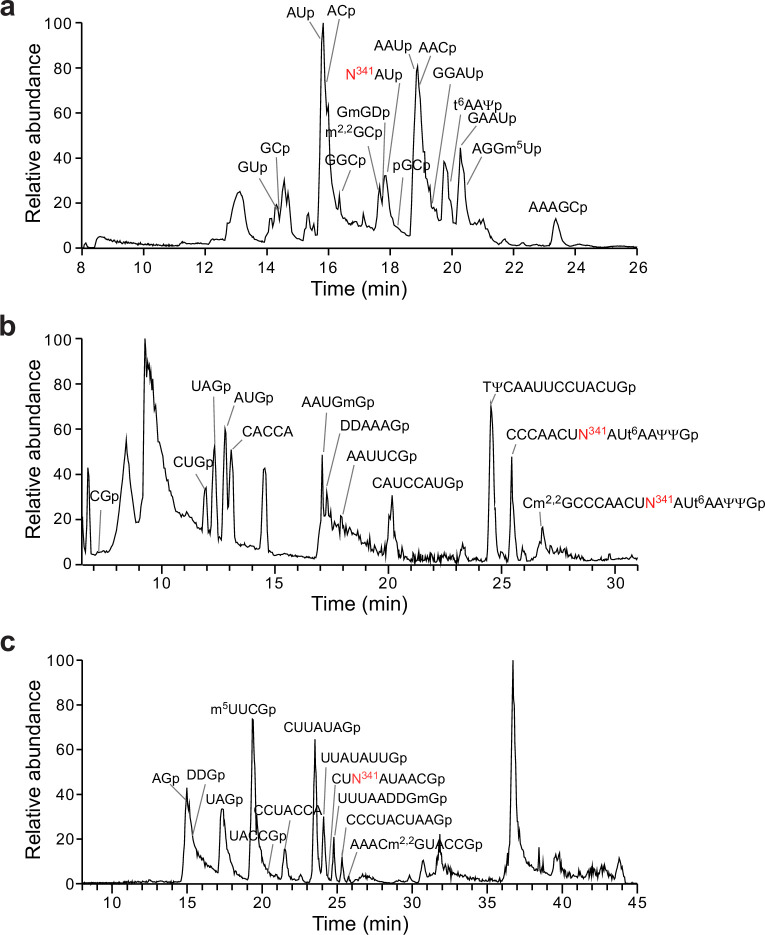
LC–MS analyses of isolated tRNAs digested by RNases. BPCs of RNA fragment analyses of spinach chloroplast tRNA^Ile2^ digested by RNase A (**a**) and RNase T_1_ (**b**), and spinach mitochondrial tRNA^Ile2^ digested by RNase T_1_ (**c**). The *m*/*z* value and charge state (*z*) of each fragment are listed in Supplementary Table 1a–c.

**Extended Data Fig. 3:**
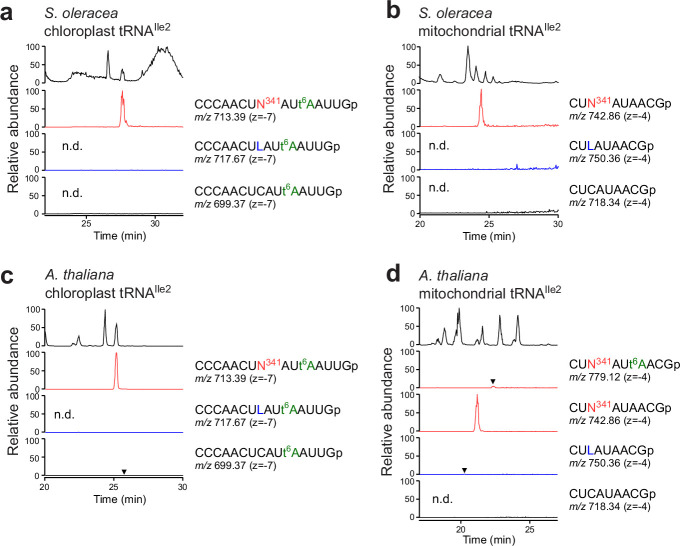
LC–MS analyses of anticodon-containing fragments. XICs of the RNase T_1_-digested fragments of tRNA^Ile2^ from spinach chloroplasts (**a**), spinach mitochondria (**b**), *A. thaliana* chloroplasts (**c**) and *A. thaliana* mitochondria (**d**). Anticodons containing fragments bearing N^341^, L or unmodified C are shown. Arrows show the target fragments. n.d., not detected.

**Extended Data Fig. 4:**
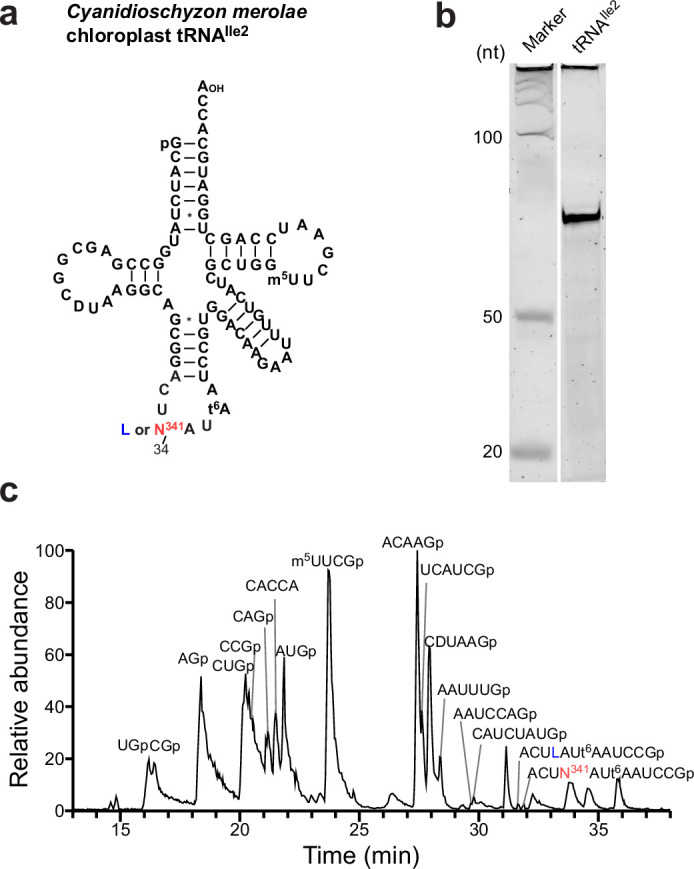
Isolation and LC–MS analysis of *C. merolae* chloroplast tRNA^Ile2^. **a**, Secondary structure of *C. merolae* chloroplast tRNA^Ile2^ including modified nucleosides. The modifications other than pseudouridine were determined in this study by LC–MS. **b**, Isolated *C. merolae* chloroplast tRNA^Ile2^ was resolved by 10% PAGE with 7 M urea, stained with SYBR Gold and visualized by a FLA-7000 scanner (Fujifilm). **c**, LC–MS analysis (BPC) of *C. merolae* chloroplast tRNA^Ile2^ digested by RNase T_1_. RNA fragments with their *m*/*z* values and charge states (*z*) are shown in Supplementary Table 1d. Source data

To determine the chemical structure of the modified nucleoside in tRNA^Ile2^ position 34 in plant organelles, tRNAs^Ile2^ from spinach (*Spinacia oleracea*) chloroplasts and mitochondria (Fig. 1a) were isolated by reciprocal circulating chromatography (RCC; Supplementary Fig. 1a,b)^31,32^, digested by RNase A and analyzed by capillary liquid chromatography (LC)–nano-electrospray ionization (ESI)–mass spectrometry (MS) to detect an anticodon-containing fragment (positions 34–36; Fig. 1b, Extended Data Fig. 2a and Supplementary Table 1a). A L-containing fragment (LAUp; *m*/*z* 1085.2; Fig. 1b) was not detected, but a fragment bearing a modified cytidine with 341 Da (*m/z* 1055.2) was observed (Fig. 1b) and tentatively named N^341^ because no RNA modified nucleoside reported to date has this mass. Collision-induced dissociation (CID) analysis revealed that N^341^ was present at position 34 (Fig. 1c). Following RNase T_1_ digestion, the anticodon-containing fragments of the tRNAs were analyzed by MS (Extended Data Figs. 2b,c, 3a,b and Supplementary Table 1b,c), which confirmed the presence of the N^341^-containing fragment and the absence of the L-containing fragment (Extended Data Fig. 3a,b). Nucleoside analyses detected a N^341^ nucleoside in total tRNA fractions from three land plants (*S. oleracea, Arabidopsis thaliana* and *Nicotiana tabacum* BY-2 cells; Fig. 1d), and no L nucleoside was detected in these samples (Fig. 1d), suggesting that organelle tRNAs^Ile2^ in land plants commonly have N^341^. Similarly, only a N^341^-containing fragment was detected in *A. thaliana* chloroplast tRNA^Ile2^ (Supplementary Fig. 1c,d and Extended Data Fig. 3c,d). In *A. thaliana* mitochondrial tRNA^Ile2^, a small amount of L was detected, but N^341^ was the major modification (Extended Data Fig. 3d). Intriguingly, we detected both N^341^ and L nucleosides in total tRNA from the unicellular red alga, *Cyanidioschyzon merolae* (Fig. 1d). To confirm this observation, we isolated chloroplast tRNA^Ile2^ from *C*. *merolae* (Extended Data Fig. 4a,b), analyzed its anticodon-containing fragments digested by RNase T_1_ (Extended Data Fig. 4c and Supplementary Table 1d) and clearly detected both N^341^- and L-containing fragments (Fig. 1e), implying some connection between N^341^ and L in terms of their molecular functions or biosynthesis.

**Extended Data Fig. 5:**
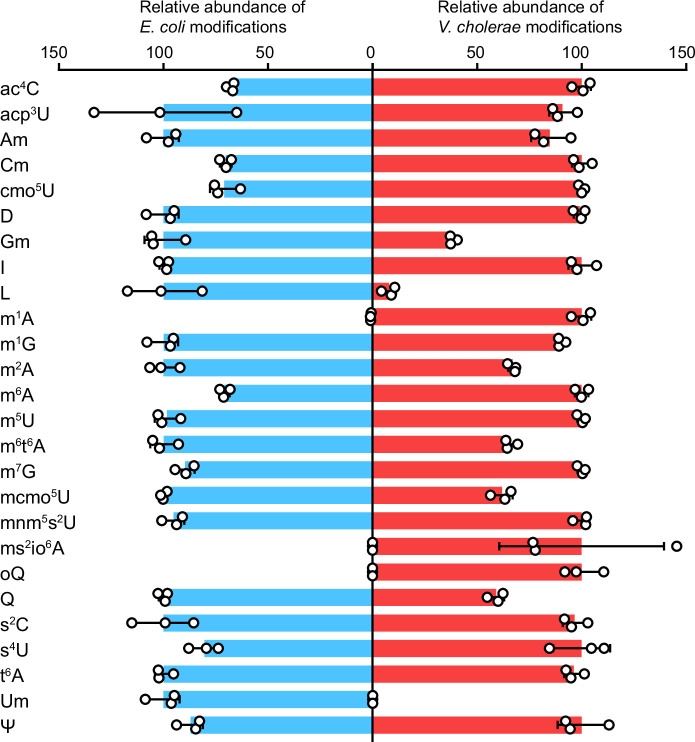
Quantification of modified nucleosides in *E. coli* and *V. cholerae* tRNAs. The relative abundances of modified nucleosides in *E. coli* and *V. cholerae* tRNAs were measured by QQQ LC–MS. In each nucleoside, an area value of mass chromatograms was normalized by a sum of the A, U, G and C area values. The higher value was set as 100%. The bars and error bars indicate the average and standard deviation from three independent cultures, respectively. Source data

**Extended Data Fig. 6:**
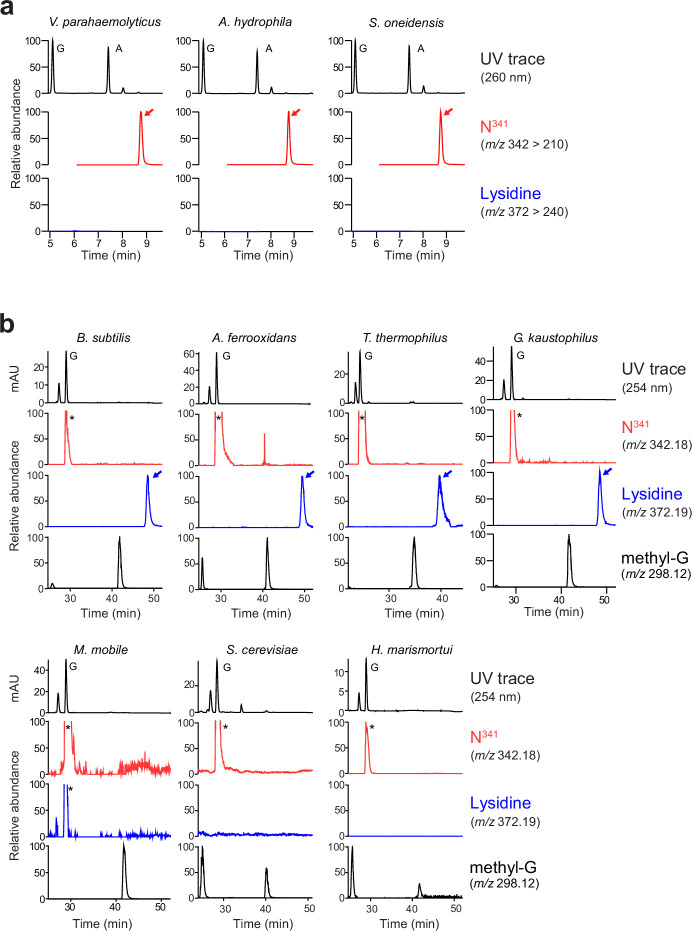
Distribution of N^341^ and L in diverse organisms. LC–MS nucleoside analyses of tRNA fractions from organisms with (**a**) and without (**b**) N^341^. The relative abundances were normalized by the highest intensity of N^341^ (**a**) or one-tenth of methyl-G (**b**) signals in each organism.

Unexpectedly, in a different project surveying tRNA modifications in certain bacteria, N^341^ was also identified in several bacterial species. Nucleoside analyses detected N^341^ and low abundance of L in the *V. cholerae* tRNAs (Fig. 1d,f and Extended Data Fig. 5). N^341^ was also observed in γ-proteobacteria, phylogenetically close to *V. cholerae*, such as *Vibrio parahaemolyticus, Aeromonas hydrophila* and *Shewanella oneidensis* (Extended Data Fig. 6a), as well as in the relatively distant species *Pseudomonas putida* (Fig. 1d). To examine whether N^341^ nucleosides from plants and bacteria are identical, we performed LC–MS co-injection analyses of spinach and *V. cholerae* total nucleosides using both normal-phase and reverse-phase column chromatographies (Fig. 1g). N^341^ nucleosides derived from two samples co-eluted as a single peak in both chromatographies (Fig. 1g), demonstrating that N^341^ derived from plants and bacteria is identical. N^341^ was not observed in *E. coli* (Fig. 1d), several other bacteria, yeast and archaeal species (Supplementary Fig. 6b). Collectively, these results demonstrate that N^341^ is a new tRNA modification distributed in plant organelles and a subset of bacterial species. The phylogenetic distribution of N^341^ and L in organisms examined here is illustrated in Supplementary Fig. 2.

### N^341^ is a modified cytidine conjugated with 5-AVA

**Fig. 2:**
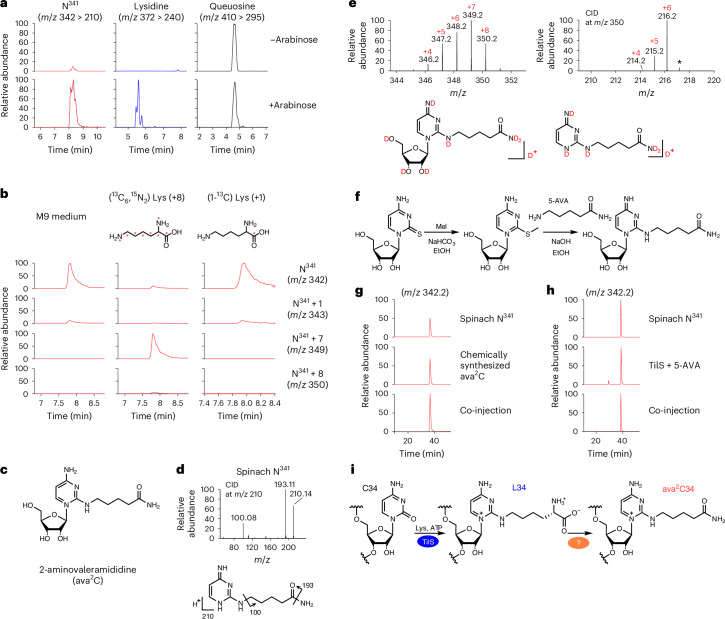
N^341^ is a modified cytidine conjugated with 5-AVA. **a**, Involvement of TilS in N^341^ biogenesis in *V. cholerae*. LC–MS analysis of total nucleosides from the *V. cholerae* strain (TilS expression is controlled by the arabinose promotor) cultured in the presence (top) or absence (bottom) of arabinose. MRM chromatograms detecting N^341^ (*m*/*z* 342 > 210), L (*m*/*z* 372 > 240) and queuosine (*m*/*z* 410 > 295) are shown. **b**, Metabolic labeling of N^341^ with stable isotope-labeled lysine. LC–MS analyses of total nucleosides from *V. cholerae* cells cultured in M9 medium (left) and M9 medium supplemented with full-labeled Lys (^13^C_6_,^15^N_2_-lysine; middle) and one carbon-labeled Lys (1-^13^C-lysine; right). XICs detecting proton adducts of N^341^ nucleoside with different isotopes as indicated on the right are shown. Asterisks represent labeled carbon and nitrogen atoms of Lys. **c**, Chemical structure (imine isomer) of ava^2^C. **d**, CID spectrum of the spinach N^341^ base moiety (BH_2_^+^) with parent *m*/*z* 210. The daughter ions are assigned on the ava^2^C base. **e**, Mass (left) and CID (right) spectra of N^341^ nucleoside in D_2_O solution. Eight and six exchangeable protons, including a proton for ionization, are assigned on the ava^2^C nucleoside (left) and its base moiety (right), respectively. The asterisk indicates a non-specific signal not derived from the D-labels. **f**, Schematic representation of the chemical synthesis of the ava^2^C nucleoside (see Methods for details). **g**, LC–MS co-injection analyses with chemically synthesized ava^2^C. Spinach N^341^ (top), chemically synthesized ava^2^C (middle) and a co-injected sample (bottom) were analyzed by an ODS column. **h**, LC–MS co-injection analyses with enzymatically synthesized ava^2^C. Spinach N^341^ (top), enzymatically synthesized ava^2^C (TilS + 5-AVA; middle) and a co-injected sample (bottom) were analyzed by an ODS column. **i**, Proposed biosynthetic pathway of ava^2^C. First, C34 is converted to L34 catalyzed by TilS using Lys and ATP as substrates. Second, L34 is further converted to ava^2^C by an unidentified enzyme(s).

N^341^ and L were abundant in *C*. *merolae* chloroplast tRNA^Ile2^ (Fig. 1e), whereas trace amounts of L were detected in tRNAs^Ile2^ from *V. cholerae* and *P. putida* (Fig. 1d), as well as *A. thaliana* mitochondria (Extended Data Fig. 3d). These observations prompted us to speculate that N^341^ is a derivative of L. To examine whether TilS is involved in the biogenesis of N^341^, we constructed a *V. cholerae* strain in which TilS expression was controlled by replacing the native promoter of the *tilS* gene with an arabinose-inducible promoter. Total nucleosides in tRNA fractions prepared from cultures grown in the presence or absence of arabinose were analyzed (Fig. 2a). N^341^ and L nucleosides were hardly detectable in the absence of arabinose (Fig. 2a), demonstrating that TilS is involved in N^341^ biogenesis. Metabolic labeling studies showed that Lys is incorporated into N^341^. *V. cholerae* was cultured in a medium supplemented with stable isotope-labeled (^13^C_6_, ^14^N_2_) Lys (+8), and total tRNA was analyzed by LC–MS (Fig. 2b). N^341^ nucleoside with a molecular mass (*m*/*z* 349) 7 Da larger than that of natural N^341^ (*m*/*z* 342) was detected, indicating that seven of eight labeled atoms of Lys were incorporated into N^341^. Because one carbon can be detached by a decarboxylation reaction, we speculated that a Lys terminal carboxy group is lost in the generation of N^341^. When we cultured *V. cholerae* in the presence of (1-^13^C) Lys (+1) whose carboxy carbon was labeled with ^13^C (Fig. 2a), only natural N^341^ nucleoside (*m*/*z* 342) was detected (Fig. 2b), demonstrating that the carboxy carbon of Lys is eliminated during biogenesis of N^341^.

The chemical structure of N^341^ was further probed by obtaining a large quantity of N^341^ nucleoside (3.26 mg; Supplementary Fig. 3a,b) from chloroplast tRNA^Ile2^ isolated by chaplet column chromatography^33^. We determined the atomic composition of N^341^ by measuring its accurate mass and found that N^341^ is a cytidine derivative with an additional mass of 98.085 Da, which predicted the chemical formula C_5_H_10_N_2_. Based on this information, N^341^ was predicted to be a cytidine derivative conjugated with 5-AVA at the C2 carbon of cytosine, which we named ava^2^C (Fig. 2c and Extended Data Fig. 1c). The CID spectrum of plant N^341^ showed good agreement with the proposed structure (Fig. 2d). Deuterium exchange with the plant N^341^ nucleoside showed there were eight hydrogens as solvent-exchangeable atoms in the N^341^ nucleoside (Fig. 2e). In addition, we analyzed a base-related ion of N^341^ by CID and found six hydrogens replaced by deuterium atoms (Fig. 2e), confirming the predicted structure.

**Extended Data Fig. 7:**
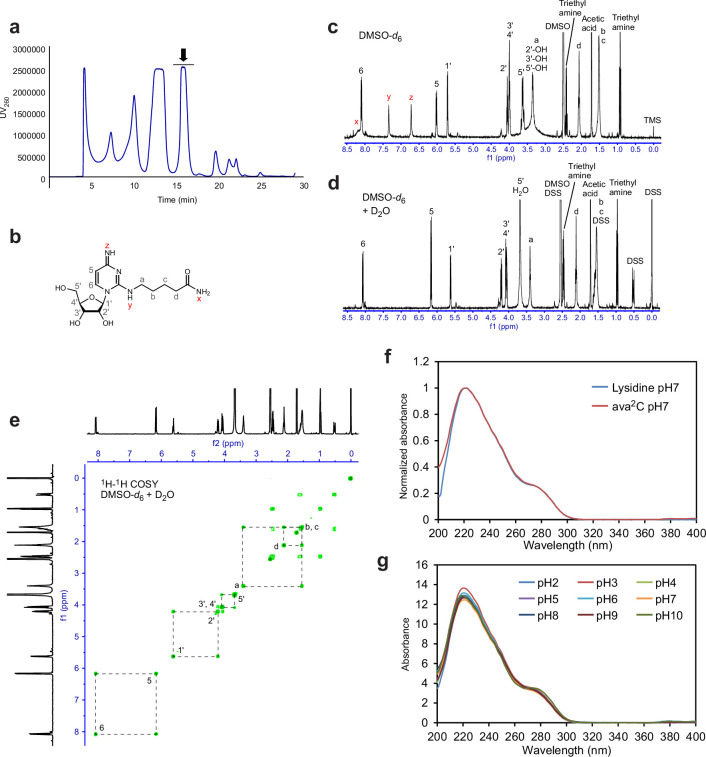
NMR and UV analyses of chemically synthesized ava^2^C. **a**, Purification of the chemically synthesized ava^2^C nucleoside by reverse-phase HPLC. The peak indicated by the arrow was collected. **b**, Chemical structure of ava^2^C. The atom names correspond to those on the NMR charts (**c**–**e**). **c**,**d**, ^1^H NMR spectra of the chemically synthesized ava^2^C nucleoside in DMSO-*d*_6_ (**c**) and DMSO-*d*_6_ + D_2_O (**d**). The chemical shifts are shown in ppm using tetramethylsilane (TMS) or solvent (DMSO-*d*_6_) as an internal standard. Signals for protons x, y and z in DMSO-*d*_6_ disappeared in DMSO-*d*_6_ + D_2_O, indicating that protons x, y and z are solvent-exchangeable. **e**, ^1^H-^1^H COSY spectrum of chemically synthesized ava^2^C in DMSO-*d*_6_ + D_2_O. Cross-peaks between the assigned protons are indicated by dashed lines. **f**, Comparison of UV spectra of ava^2^C and L. The spectra were normalized at the maximum absorption wavelength. The two nucleosides showed almost identical spectra. **g**, UV spectra of synthetic ava^2^C in different pH solutions. Specifically, 50 mM sodium phosphate buffer (pH 2, 3 and 6–9), sodium acetate buffer (pH 4 and 5) and sodium borate buffer (pH 10) were used. Source data

To validate the chemical structure of N^341^, an authentic ava^2^C nucleoside was chemically synthesized following the scheme shown in Fig. 2f. The final product was purified by HPLC (Extended Data Fig. 7a) and analyzed by nuclear magnetic resonance (NMR) to confirm the chemical structure (Extended Data Fig. 7b–e). In the ^1^H NMR spectrum of synthetic ava^2^C in deuterated dimethyl sulfoxide (DMSO-d_6_), all the protons in the ava^2^C base were assigned, including 5(CH), 6(CH), x(NH_2_), y(NH), z(NH), a(CH2), b(CH2), c(CH2) and d(CH2), as well as all the protons in ribose (H3′, H2′, H3′, H3′, H3′, 2′-OH, 3′-OH and 3′-OH; Extended Data Fig. 7b,c). In the presence of D_2_O, all solvent-exchangeable protons of the ava^2^C base, x(NH_2_), y(NH) and z(NH), disappeared as expected (Extended Data Fig. 7b,d). By tracing the cross-peaks in the ^1^H-^1^H correlation spectroscopy (COSY) spectrum (Extended Data Fig. 7e), all assigned protons were connected as expected by the chemical structure of ava^2^C. These results confirmed the chemical structure of the synthetic ava^2^C. The ultraviolet spectrum of ava^2^C was almost identical to that of L (Extended Data Fig. 7f,g), indicating that the ring moiety of ava^2^C has similar characteristics to L^18^. An LC–MS co-injection analysis of the chemically synthesized ava^2^C and plant N^341^ revealed that both nucleosides eluted at the same retention time as a single peak (Fig. 2g). We also synthesized ava^2^C by an enzymatic reaction with TilS (Supplementary Fig. 4a). Because TilS has low substrate specificity for Lys^34^, it is possible to incorporate a broad range of Lys analogs into tRNA using TilS in vitro. We successfully introduced ava^2^C into tRNA^Ile2^ using *E. coli* TilS in the presence of a high concentration (10 mM) of 5-AVA (Supplementary Fig. 4a). We performed the co-injection analysis and confirmed that the TilS-synthesized ava^2^C co-eluted with plant N^341^ as a single peak (Fig. 2h). In addition, the CID spectrum of N^341^ nucleoside was identical to those of the synthesized ava^2^Cs (Supplementary Fig. 5). We conclude that both plant and bacterial N^341^ is ava^2^C.

Because *E. coli* TilS artificially synthesized ava^2^C with 5-AVA in vitro (Supplementary Fig. 4a), we investigated whether *V. cholerae* TilS directly synthesizes ava^2^C using 5-AVA as a metabolic substrate. To this end, a small compound fraction (metabolites) extracted from *V. cholerae* was incubated with unmodified tRNA^Ile2^ and *V. cholerae* or *E. coli* recombinant TilS (Supplementary Fig. 6a,b) and then analyzed by LC–MS. Only L and no ava^2^C were detected in both cases (Supplementary Fig. 6c), demonstrating that *V. cholerae* TilS uses Lys to synthesize L and suggesting that an additional enzyme(s) converts L to ava^2^C (Fig. 2i).

### ava^2^C facilitates AUA decoding

**Fig. 3:**
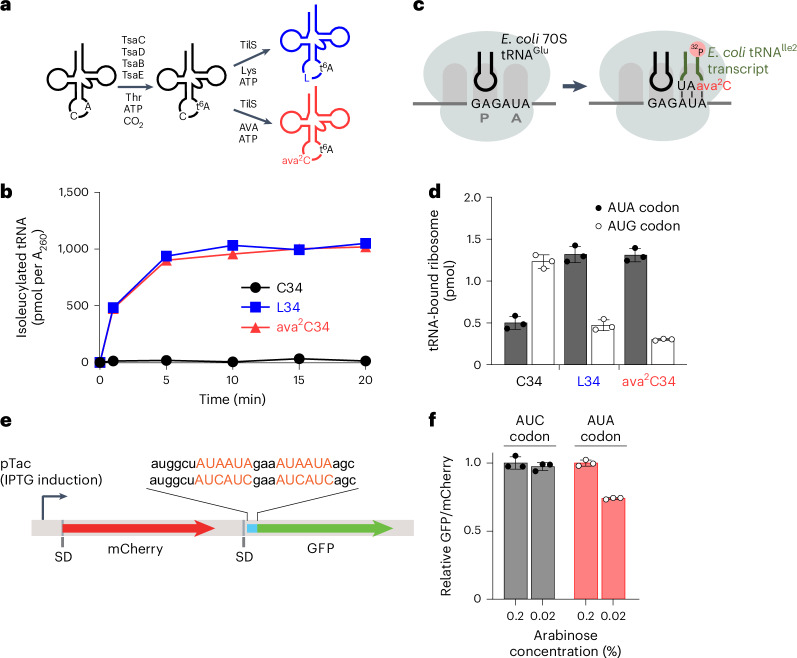
ava^2^C promotes AUA decoding in translation. **a**, Schematic representation of the enzymatic synthesis of *E. coli* tRNA^Ile2^ transcripts bearing t^6^A37 and L34 or ava^2^C34. **b**, In vitro isoleucylation of tRNA transcripts bearing C34 (circles), L34 (rectangles) or ava^2^C34 (triangles). **c**, Schematic depiction of tRNA binding to the A-site of the *E. coli* ribosome. The P-site was occupied by *E. coli* tRNA^Glu^, followed by incubation with ^32^P-labeled tRNA transcripts with different modification statuses. **d**, Ribosome-binding ability of *E. coli* tRNA^Ile2^ transcripts bearing C34, L34 or ava^2^C34 to examine decoding of the AUA (filled bars) or AUG (blank bars) codon at the A-site. The background signal measured in the same reaction without mRNA was subtracted. Data are shown as mean ± s.d. (*n* = 3 technical replicates). **e**, Constructs of dual reporters to measure the decoding efficiency of Ile codons (AUA or AUC) in the *V. cholerae* strain. Ile codons (AUA or AUC) were tandemly inserted at the N-terminus of GFP. GFP signals normalized by mCherry signals reflect the translation efficiency of Ile codons. **f**, Dual reporter assay evaluating the decoding efficiency of AUC or AUA codons in the *V. cholerae* strain cultured with 0.2% or 0.02% arabinose. Relative values of GFP/mCherry are shown as mean ± s.d. (*n* = 3 biological replicates). A multiple two-tailed *t* test was used for a statistical test. Comparisons are made between arabinose 0.2% and 0.02%. *P* = 0.44 (AUC reporter) and *P* = 0.000078 (AUA reporter) after adjustment by the Holm test. Source data

We next studied the roles of ava^2^C in protein synthesis. In vitro transcribed tRNA^Ile2^ containing *N*^6^-threonylcarbamoyladenosine (t^6^A) at position 37 was prepared by enzymatic reconstitution (Fig. 3a and Supplementary Fig. 4b) because t^6^A37 is a strong positive determinant for both lysidylation and isoleucylation^35^. Then, ava^2^C or L was introduced into the tRNA at position 34 by TilS (Fig. 3a and Supplementary Fig. 4b). We first examined the Ile-accepting ability of the tRNA with or without ava^2^C34 by *E. coli* IleRS (Fig. 3b). As controls, the L34-containing tRNA^Ile2^ was efficiently charged with Ile, whereas the tRNA^Ile2^ with unmodified C34 exhibited no isoleucylation. The ava^2^C34-containing tRNA^Ile2^ was charged with Ile as efficiently as the L34-containing tRNAs, indicating that ava^2^C34 promotes charging by IleRS. To assess the impact of ava^2^C34 on AUA decoding, we conducted a nonenzymatic A-site tRNA-binding experiment (Fig. 3c)^27^. As controls, tRNA^Ile2^ with unmodified C34 efficiently bound the AUG codon but not the AUA codon (Fig. 3d), whereas tRNA^Ile2^ with L34 specifically recognized the AUA codon (Fig. 3d). tRNA^Ile2^ with ava^2^C34 specifically bound the AUA codon (Fig. 3d), suggesting that tRNA^Ile2^ acquired the ability to decode the AUA codon via ava^2^C formation. Together, these results demonstrate that ava^2^C controls tRNA^Ile^2′s capacity to be aminoacylated and to bind the AUA codon.

We used a dual-reporter assay to examine the role of ava^2^C in AUA decoding in the cell. Two sets of consecutive Ile codons (AUA or AUC) were inserted at the beginning of the GFP gene in a construct that also included a gene encoding mCherry (Fig. 3e). The decoding efficiency of the AUA codon was evaluated by measuring GFP signals normalized by mCherry signals. These constructs were introduced into a *V. cholerae* strain where TilS expression was controlled by an arabinose-inducible promotor. Upon TilS depletion in low (0.02%) arabinose, the ratio of GFP to mCherry signals in the AUA reporter decreased markedly (Fig. 3f), whereas no decrease was observed in the AUC reporter (Fig. 3f), demonstrating that ava^2^C promotes AUA decoding in the cell.

### Structural basis of AUA decoding by ava^2^C34

**Fig. 4:**
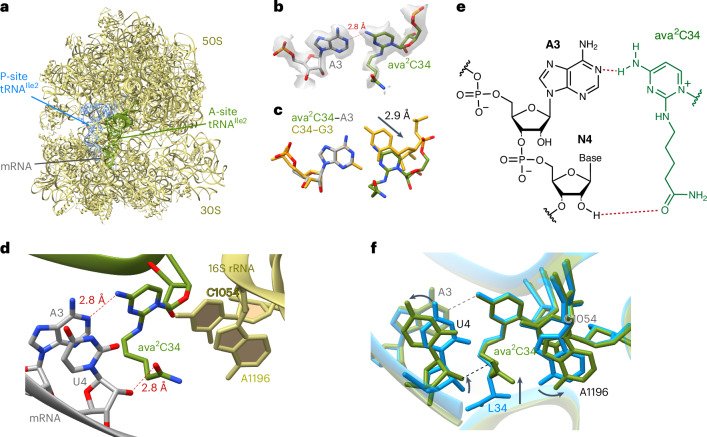
Cryo-EM structural analysis of AUA decoding by ava^2^C34. **a**, Atomic model of the *E. coli* 70S ribosome bound with mRNA (gray) and *P. putida* tRNAs^Ile2^ at the P-site (blue) and A-site (green). **b**, Base pairing geometry of ava^2^C34 recognizing A3 of the AUA codon. The cryo-EM map (contoured at level 0.0277) is superimposed on the atomic models. **c**, Comparison of base pairing geometries between C34–G3 (orange, PDB ID: 4V5R)^39^ and ava^2^C34–A3 (green). The cytosine of ava^2^C34 moves to its minor groove by 2.9 Å compared with the C34–G3 pair. **d**, Model structures of ava^2^C34 recognizing the AUA codon in the decoding center of the A-site. Potential hydrogen bonds and the distances are indicated by dotted lines and red text, respectively. **e**, Chemical structures of ava^2^C34 recognizing the AUA codon facilitated by the hydrogen bond between the amide group of ava^2^C and 2′-OH of the mRNA residue 3′-adjacent to the AUA codon. The carbonyl oxygen of ava^2^C forms a hydrogen bond with 2′-OH of N4 residue in this model. Potential hydrogen bonds are shown by dotted lines. **f**, Structural comparison of ava^2^C34–A3 (green) and L34–A3 (blue) pairs at the A-site. Rotation and movement of U4 in mRNA and A1196 in 16S rRNA between these two structures are illustrated by arrows. Hydrogen bonds are shown by dotted lines.

**Extended Data Fig. 8:**
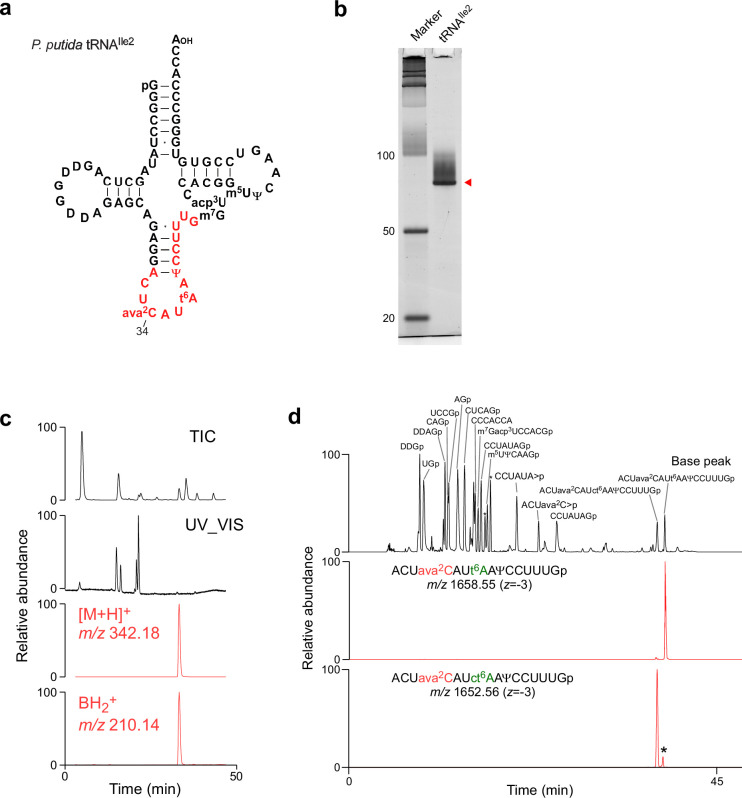
Isolation and LC–MS analysis of *P. putida* tRNA^Ile2^. **a**, Secondary structure of *P. putida* tRNA^Ile2^ including modified nucleosides determined in this study by LC–MS. The RNase T_1_-digested fragment containing the anticodon is highlighted in red. **b**, Isolated *P. putida* tRNA^Ile2^ was resolved by 10% PAGE with 7 M urea, stained with SYBR Gold and visualized by a FLA-7000 scanner (Fujifilm). **c**, LC–MS nucleoside analysis of the isolated tRNA. The total ion chromatogram (TIC; top), UV trace (second) and mass chromatograms of ava^2^C nucleoside (M + H)^+^ (*m*/*z* 342.18, third) and its base-related ion BH_2_^+^ (*m*/*z* 210.14, bottom) are shown. **d**, LC–MS analysis of *P. putida* tRNA^Ile2^ digested by RNase T_1_. The BPC (top) and XICs of the anticodon-containing fragments bearing ava^2^C34 and t^6^A37 (middle) and ava^2^C34 and ct^6^A37 (bottom) are shown. RNA fragments with their *m*/*z* values and charge states (*z*) are shown in Supplementary Table 1e. A nonspecific peak with the same *m*/*z* was marked with an asterisk. Source data

**Extended Data Fig. 9:**
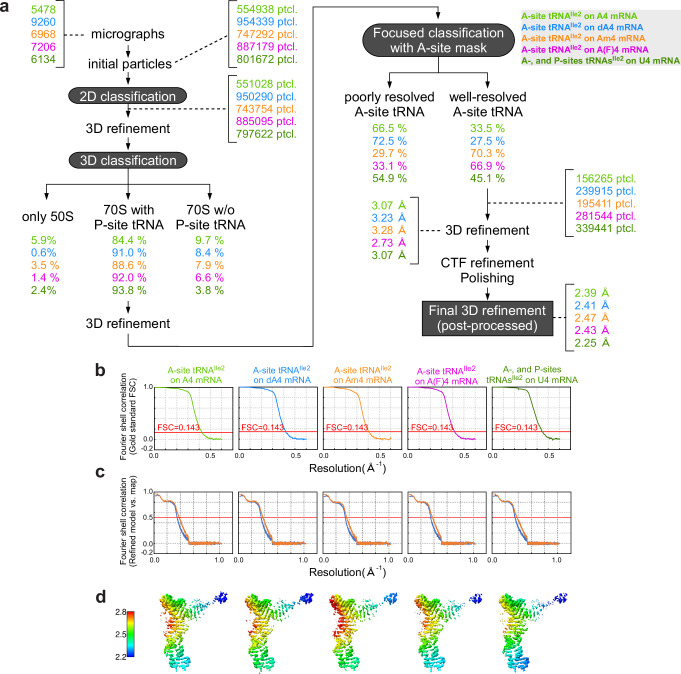
Cryo-EM image processing. **a**, Scheme of cryo-EM image processing, which was performed in parallel for five 70S complexes described in this study. After 3D auto-refinement and 3D classification, the subclasses of 70S ribosomes with P-site tRNA density were pooled for 3D refinement to include all particles potentially bound with *P. putida* tRNA^Ile2^ at the A-site. Focused classification was performed using an A-site mask to generate the final map of the complex occupied by tRNAs at both the A- and P-sites. **b**,**c**. Fourier shell correlation curves of the complexes (**b**) and models vs. cryo-EM maps (**c**). From left to right, *P. putida* tRNA^Ile2^ on A4 mRNA, dA4 mRNA, Am4 mRNA and A(F)4 mRNA, and A- and P-sites *P. putida* tRNAs^Ile2^ on U4 mRNA. **d**, Cryo-EM densities of tRNA and mRNA codon at A-site extracted from the ribosome complex corresponding to **b** and **c**. Each map is colored according to the local resolution, and the color key is drawn on the left.

To understand the molecular basis by which ava^2^C contributes to efficient and specific recognition of the AUA codon, we performed single-particle cryo-EM analysis of the ribosome complexed with tRNA and mRNA. We isolated tRNA^Ile2^ from *P. putida* (Extended Data Fig. 8a,b), analyzed its modification status by LC–MS (Extended Data Fig. 8c,d) and confirmed a high frequency of ava^2^C at position 34. Given the high conservation of the decoding center across species^36^, we used a combination of tRNA and ribosomes from distinct sources to prepare cryo grids—*P. putida* tRNA^Ile2^ was bound to both P- and A-sites of the *E. coli* 70S ribosome. To extract a class of complexes with two tRNAs occupying both the A- and P-sites, we applied focused classification^37^ with subregional signal subtraction from the ribosome particles (Extended Data Fig. 9a). Finally, we solved a cryo-EM structure of the *E. coli* 70S ribosome with two *P. putida* tRNAs^Ile2^ at both the A- and P-sites in the classical state at 2.25 Å resolution (Fig. 4a, Extended Data Fig. 9a–d and Supplementary Table 2). This structure represents the closed conformation of the 30S subunit in which A1492, A1493 and G530 of 16S rRNA interact with the minor groove of the codon–anticodon duplex^38^.

**Extended Data Fig. 10:**
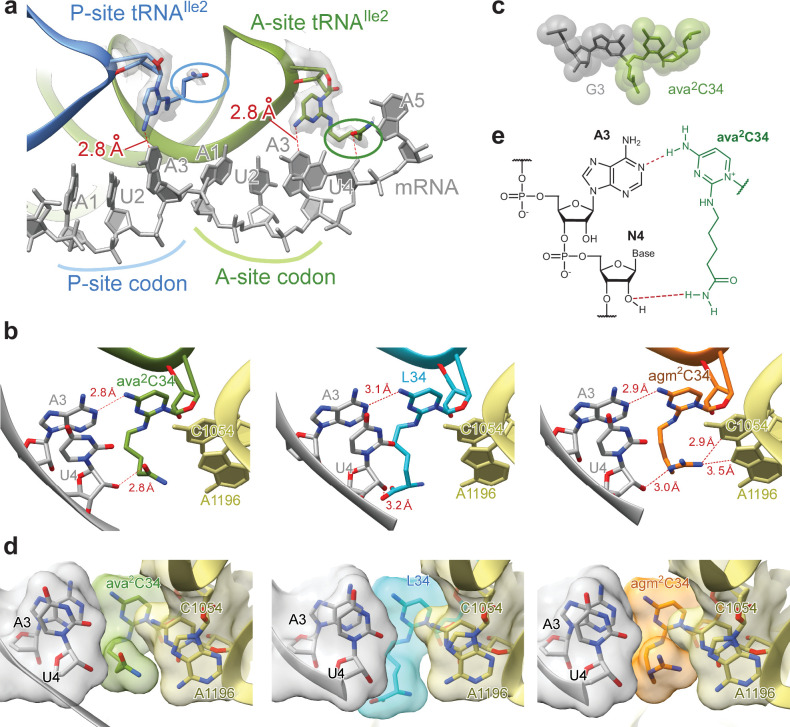
Structural characterization of the ava^2^C–A pair on the ribosome and its comparison with other cytidine modifications. **a**, Codon–anticodon interactions at P- and A-sites of the 70S ribosome. mRNA kinks by ~45° between P- and A-site codons. ava^2^C–A pairs are visible and common at both sites, but the density of the ava^2^C side chains is seen only at the A-site. **b**, Model structures of ava^2^C34 (left), L34 (middle) and agm^2^C34 (right) recognizing the AUA codon in the decoding center of the A-site. Potential H-bonds and the distances are indicated by dotted lines and red text, respectively. **c**, Hypothetical ava^2^C34–G3 pairing in a canonical Watson–Crick geometry. The aminovaleramide group of ava^2^C34 clashes with N_2_-amine of G3. **d**, Solvent-excluded surface models showing the structural complementarity of the long side chains of ava^2^C34 (left), L34 (middle) and agm^2^C (right), and the cleft formed by rRNA residues and the mRNA strand at the A-site. A large area of van der Waals contacts between ava^2^C34 and rRNA residues, and the mRNA is clearly visible. **e**, Another possible hydrogen bonding pattern of ava^2^C34 and mRNA residues.

In the codon–anticodon helix, ava^2^C34 pairs with the third adenine of the AUA codon via a single hydrogen bond at both the A- and P-sites (Fig. 4b and Extended Data Fig. 10a). This geometry is identical to those observed in the C34–A3 pair in Hirsh suppressor tRNA^Trp^ (refs. ^39,40^), the agm^2^C34–A3 pair in archaeal tRNA^Ile2^ (refs. ^27,41^) and the L34–A3 pair in bacterial tRNA^Ile2^ (refs. ^27,28^; Extended Data Fig. 10b). When we compare the base pairing geometry between ava^2^C34–A3 pair and C34–G3 Watson–Crick pair, ava^2^C34 moves toward its minor groove by 2.9 Å (Fig. 4c). This geometry explains why ava^2^C34 is unable to pair with G3 of the AUG codon; the aminovaleramide group of ava^2^C34 causes steric clashes with the N_2_-amine of G3 in Watson–Crick geometry (Extended Data Fig. 10c). Otherwise, upon binding to the AUG codon, ava^2^C34 might facilitate unusual Hoogsteen base pairing with G3 as observed in the L34–G3 pair at the A/T state of the 70S ribosome complex^28^, thereby rejecting the EF–Tu ternary complex. Thus, ava^2^C avoids misreading the AUG codon and ensures AUA decoding due to the unique base pairing property conserved with other modified cytidines, L and agm^2^C.

Because the C–A geometry mediated by a single hydrogen bond is thermodynamically unstable, the side chains of both L34 and agm^2^C34 extend downstream of the mRNA, and the terminal polar groups form an additional hydrogen bond with 2′-OH of the residue 3′-adjacent to the AUA codon^27^. At the A-site (Fig. 4b), we observed a clear density of the aliphatic group of ava^2^C34 side chain, which extends toward the 3′ direction of mRNA and fills in the space surrounded by G530, C1054 and A1196 of 16S rRNA and the fourth mRNA residues (Fig. 4d and Extended Data Fig. 10b,d). Although the density of the terminal amide group of ava^2^C34 is relatively weak, the amide group plausibly forms a hydrogen bond with the 2′-OH of the uridine (U4) 3′-adjacent to the AUA codon (Fig. 4d,e). Two rotamers of ava^2^C34 would be equally possible to make a hydrogen bond via the carbonyl (Fig. 4e) or amino (Extended Data Fig. 10e) group.

Compared with the L34 structures^27^, the U4 residue in the ava^2^C structure is slightly rotated toward the major groove (Fig. 4f). The terminal amide group of ava^2^C is oriented in a position that pushes U4 because ava^2^C has a side chain one atom shorter than those of L and agm^2^C (Extended Data Fig. 1a–c). This rotation slightly moves the 2′-OH of U4 toward the amide group of ava^2^C in a hydrogen bond distance (Fig. 4f). In addition, the amide group moves closer to the rRNA residues, leading to the obvious shift of A1196 (Fig. 4f).

At the P-site (Extended Data Fig. 10a), no clear density of the side chain of ava^2^C34 is seen because the additional hydrogen bond cannot form between the amide group of ava^2^C34 and the 3′-adjacent residue of the P-site codon, which is the first letter of the A-site codon. The mRNA strand kinks by ~45° between P- and A-site codons^42,43^. The residue 3′-adjacent to the P-site codon is not within a hydrogen bond distance of the ava^2^C34 amide group (Extended Data Fig. 10a).

### Characterization of AUA decoding with modified mRNAs

**Fig. 5:**
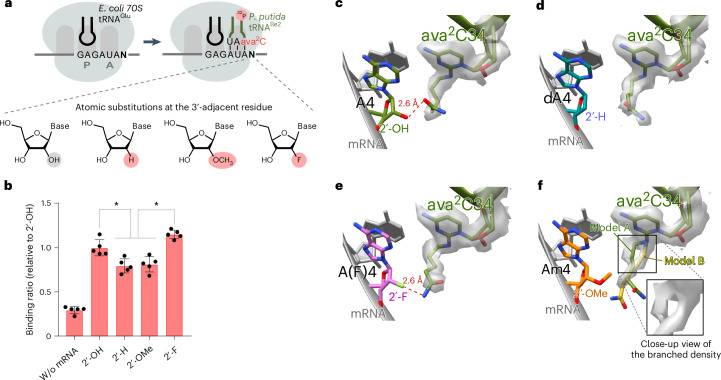
Characterization of AUA decoding by ava^2^C34 with synthetic mRNAs. **a**, Schematic depiction of tRNA binding to the A-site of the *E. coli* ribosome using synthetic mRNAs with atomic substitutions at the fourth nucleotide (N)—unmodified (2′-OH, left), 2′-H (second), 2′-*O*-methyl nucleoside (2′-OMe, third) and 2′-F (right). The P-site was occupied by *E. coli* tRNA^Glu^, followed by incubation with ^32^P-labeled *P. putida* tRNA^Ile2^ to examine AUA decoding upon 2′-OH substitutions at the fourth residue. **b**, Ribosome-binding ability of *P. putida* tRNA^Ile2^ on the AUA codon of mRNAs with different 2′-OH substitutions at the fourth residue, namely, unmodified (2′-OH), 2′-H, 2′-OMe and 2′-F. Nonspecific tRNA binding was estimated without (w/o) mRNA. The binding ratio was calculated as the ratio of bound tRNA to input tRNA. Data are shown as mean ± s.d. (*n* = 5 technical replicates). Tukey’s adjusted *P* values are as follows: *P* = 0.002 (2′-OH versus 2′-H), *P* = 0.0048 (2′-OH versus 2′-OMe) and *P* < 0.0001 (2′-H versus 2′-F and 2′-OMe versus 2′-F). *P < 0.05. **c**–**f**, Comparison of base pairing geometry and side chain orientation of ava^2^C34 upon binding to the AUA codon with different 2′-OH substitutions at the fourth residue—A4 (2′-OH, **c**), dA4 (2′-H, **d**), A(F)4 (2′-F, **e**) and Am4 (2′-OMe, **f**). On interacting with Am4 mRNA, the ava^2^C side chain represents the branched density, to which models A (green) and B (yellow) are assigned. Source data

To examine the effect of the additional hydrogen bonding mediated by the ava^2^C side chain on AUA decoding, we performed an A-site tRNA-binding experiment using a series of synthetic mRNAs bearing 2′-OH (control), 2′-H, 2′-OMe or 2′-F at the residue 3′-adjacent to the A-site codon (Fig. 5a). *P. putida* tRNA^Ile2^ bound the AUA codon less efficiently in mRNAs with 2′-H and 2′-OMe than in the control mRNA (2′-OH; Fig. 5b), likely because 2′-H substitution loses hydrogen bonding ability and 2′-OMe substitution may hinder hydrogen bonding due to its bulkiness. By contrast, the binding efficiency of the AUA codon was unaffected by 2′-F substitution (Fig. 5b) because 2′-F acts as a hydrogen bond acceptor. These findings are consistent with our structural observation that the ava^2^C terminal amide group makes an additional hydrogen bond with 2′-OH of the 3′-adjacent residue in the mRNA (Fig. 4d,e and Extended Data Fig. 10e), ensuring stable recognition of the AUA codon at the A-site.

To further characterize AUA decoding by ava^2^C34 with the synthetic mRNAs, we solved the cryo-EM structures of 70S ribosomes complexed with *P. putida* tRNA^Ile2^ and synthetic mRNAs bearing 2′-H, 2′-OMe or 2′-F at the residue 3′-adjacent to the A-site codon (Extended Data Fig. 9a–d and Supplementary Table 2). The orientation of the ava^2^C side chain remains unchanged upon binding to mRNAs bearing 2′-H and 2′-F (Fig. 5c–e), confirming that these atomic mutations do not have any steric effect but alter the chemical properties of the 3′-adjacent residues. As expected, the amide group of ava^2^C34 is placed in a hydrogen bond distance of 2′-F of the synthetic mRNA (Fig. 5e). By contrast, we observed an aberrantly branched density of the aminovaleramide group of ava^2^C34 when bound to the synthetic mRNA with the 2′-OMe substitution (Fig. 5f). This density was assigned to two conformers of ava^2^C34 in different orientations—model A is similar to the canonical configuration of ava^2^C bound to the unmodified mRNA (2′-OH; Fig. 5c), while model B represents an alternative configuration presumably caused by the steric clash with the bulky 2′-OMe group of the mRNA (Fig. 5f). Consequently, the 2′-OMe substitution would perturb the canonical configuration of ava^2^C and weakens the interaction between ava^2^C34 and the 3′-adjacent residue of mRNA, thereby reducing AUA decoding by ava^2^C34 (Fig. 5b). We conclude that the side chain of ava^2^C34 forms an additional hydrogen bond to compensate for the unstable C–A pair to ensure efficient AUA decoding.

## Discussion

Here we discovered ava^2^C, a new cytidine modification, in tRNA^Ile2^ isolated from plant organelles and certain bacteria. ava^2^C is an L derivative whose carboxy group is replaced by a carbonyl group. ava^2^C enables tRNA^Ile2^ to be charged with Ile and to recognize the AUA codon, suggesting that this single tRNA modification governs both codon and amino acid specificities. Along with L and agm^2^C, ava^2^C is the third modified cytidine found in tRNA^Ile2^ responsible for AUA decoding.

We propose two possible biosynthetic pathways for ava^2^C. One pathway involves ava^2^C formation in two consecutive reactions. One pathway is a two-step reaction in which TilS first conjugates Lys to C34 of tRNA^Ile2^ to form L34, and then an unknown enzyme(s) converts the carboxyl group of L34 into a carbonyl group to form ava^2^C34 (Fig. 2i). The other pathway is a one-step reaction in which TilS directly synthesizes ava^2^C34 by introducing 5-AVA. Due to its low substrate specificity, TilS might introduce a broad range of Lys analogs into tRNA in vitro^34^. In this study, we artificially synthesized ava^2^C in tRNA^Ile2^ by TilS in the presence of a high concentration of 5-AVA. If the substrate specificity of TilS is changed from Lys to 5-AVA, the latter pathway is possible. Our finding that *V. cholerae* TilS forms L34, not ava^2^C34, in the presence of cell metabolites may favor the two-step model; however, our efforts to identify a second enzyme required for the biogenesis of ava^2^C34 were unsuccessful. Metabolic labeling revealed that the ava^2^C side chain originates from Lys and that the carboxyl carbon of Lys is lost in ava^2^C biogenesis. Given that ava^2^C34 and L34 coexist in *C. merolae* chloroplast tRNA^Ile2^ and that a trace amount of L is present in *A. thaliana* mitochondrial tRNA^Ile2^ and tRNA fractions from *V. cholerae* and *P. putida*, it is reasonable to propose that ava^2^C is generated from L. Identification of the enzyme(s) that converts L to ava^2^C will provide much insight into the evolutionary and phylogenetic distribution of ava^2^C.

RASPBERRY3 (RSY3) is a plant homolog of bacterial *tilS*^44^, suggesting that ava^2^C biogenesis relies on RSY3 in plants. RSY3 is thought to localize to chloroplasts, and knockout of this gene results in embryonic lethality with abnormal chloroplast development^44^. The possible link between RSY3 and ava^2^C formation implies that ava^2^C has an essential role in the embryogenesis of *A. thaliana*. According to subcellular localization prediction^45^, RSY3 localizes to both mitochondria and chloroplasts, indicating that both mitochondrial and chloroplast tRNAs^Ile2^ might be modified by RSY3 to form ava^2^C34.

In this study, we solved the cryo-EM structure of *E. coli* ribosome complexed with *P. putida* tRNA^Ile2^ and an AUA codon at the A-site in the classical A/A state. It is important to note that decoding occurs in the A/T state, where the ribosome is bound by a ternary complex composed of EF–Tu, GTP and aminoacyl-tRNA. Because the A/A state represents a postdecoding and postaccommodation phase, it would typically be more insightful to resolve the codon–anticodon pairing in the A/T state. However, in this study, we prepared a ribosome complex in the A/A state via nonenzymatic tRNA binding due to technical limitations. Nevertheless, it is established that the codon–anticodon interactions are essentially identical in both states^46^. Recent studies demonstrated that the L34 side chain remains in the same position at the A-site across both the A/T and A/A states^27,28^. Therefore, we opted to solve the cryo-EM structure of the A/A state instead of the A/T state.

The geometry of the base pairing showed that the cytosine of ava^2^C largely moves toward the minor groove to form a unique C–A pair with a single hydrogen bond. In addition, the ava^2^C side chain extends to the 3′ side of the AUA codon to fill in the cleft between rRNA residues and the mRNA strand. This interaction stabilizes the ava^2^C–A base pairing by excluding solvent from the cleft and making a van der Waals interaction with mRNA and rRNA residues. Moreover, the terminal amide group forms a hydrogen bond with the 2′-OH of the residue 3′-adjacent to the AUA codon. Indeed, biochemical and structural analyses using synthetic mRNAs showed that this additional hydrogen bond supports efficient recognition of the AUA codon. Although this hydrogen bond only involves the ribose moiety of the mRNA residue, it is plausible to speculate that the mRNA residue 3′-adjacent to the AUA codon could influence AUA decoding, because the base of the mRNA residue can engage in stacking interaction with the AUA codon. Further study is necessary to elucidate whether AUA decoding is modulated by the 3′-adjacent residue in a context-dependent manner.

According to recent studies^27,28^, the base pairing of L–A and agm^2^C–A exhibits the same geometry as the ava^2^C–A pair (Extended Data Fig. 10b). The side chains of L and agm^2^C also extend to the 3′-side of the AUA codon and occupy the space between the rRNA and mRNA (Extended Data Fig. 10d). Their terminal polar residues form a hydrogen bond with the 2′-OH of the residue 3′-adjacent to the AUA codon (Extended Data Fig. 10b). However, the orientation of the side chains differs between these three modified cytidines (Extended Data Fig. 10b,d). The L side chain is oriented toward the mRNA, and the terminal carboxy or amino group forms a hydrogen bond with the 2′-OH of the 3′-adjacent residue. The terminal guanidino group of agm^2^C is positioned between the mRNA and rRNA and forms hydrogen bonds with the 2′-OH of the 3′-adjacent residue of mRNA as well as with C1054 and A1196 of 16S rRNA, thereby establishing a more stable interaction. In contrast, the ava^2^C side chain adopts a different orientation with the terminal amide group interacting with the residue 3′-adjacent to the AUA codon because the side chain of ava^2^C is one atom shorter than those of L and agm^2^C (Extended Data Fig. 1a–c). This interaction slightly repositions the base of the residue toward its major groove so that its 2′-OH moves toward the amide group of ava^2^C to form a hydrogen bond. Moreover, the amide group comes closer to the rRNA residues to slightly move A1196; that is, the position of the 3′-adjacent residue of the mRNA and the terminus of the ava^2^C side chain is coordinated. These observations indicate that the ava^2^C side chain has better steric complementarity to the cleft between the rRNA and mRNA than the L side chain (Extended Data Fig. 10d). In fact, the cryo-EM density of the L side chain is rather weak^27^, but that of the ava^2^C side chain is clear, suggesting that the ava^2^C side chain is less flexible and the interaction of ava^2^C is stronger than that of L. Unique to ava^2^C, the terminal amide group is chemically stable without any charge. ava^2^C might have been acquired during evolution as a more stable and functional derivative of L for efficient translation of the AUA codon. Our discovery of ava^2^C provides another compelling illustration of how evolutionary innovations in the chemistry of tRNA modifications can optimize decoding.

## Methods

### Strains and medium

The organisms used in this study are listed in Supplementary Table 3. Antibiotics were used in the following concentrations: 200 μg ml^−1^ streptomycin, 50 μg ml^−1^ carbenicillin, 50 μg ml^−1^ kanamycin (Km) and 1 μg ml^−1^ chloramphenicol (Cm).

*E. coli* strain BW25113 was cultured in LB medium at 37 °C for 18 h. *Bacillus subtilis* strain 168 was cultured in LB medium at 37 °C for 24 h. *P. putida* NITE Biological Resource Center (NBRC) 14164, *Geobacillus kaustophilus* NBRC 102445 and *Acidimicrobium ferrooxidans* DSM 10331 were obtained from NBRC. *P. putida* was cultured in NBRC 702 medium (10 g l^−1^ tryptone, 2 g l^−1^ yeast extract and 1 g l^−1^ MgSO_4_·7H_2_O) at 30 °C for 22.5 h. *G. kaustophilus* was cultured in NBRC 702 medium at 55 °C for 23 h. *A. ferrooxidans* was cultured in 0.5 g l^−1^ MgSO_4_·7H_2_O, 0.4 g l^−1^ (NH_4_)_2_SO_4_, 0.2 g l^−1^ K_2_HPO_4_, 0.1 g l^−1^ KCl, 10 mg l^−1^ FeSO_4_·7H_2_O and 0.25 g l^−1^ yeast extract (adjusted to pH 2.0 with 2 M H_2_SO_4_) at 45 °C. *Mycoplasma mobile*, kindly provided by M. Miyata (Osaka City University), was grown in Aluotto medium (pH 7.8), which was composed of 2.1% heart infusion broth (Difco), 0.56% yeast extract, 10% horse serum (inactivated at 56 °C) and 0.005% ampicillin at 25 °C. Cell pellets of *Haloarcula marismortui* were kindly provided by T. Fujiwara (Shizuoka University). *V. cholerae* and *V. parahaemolyticus* were cultured in LB or M9 medium at 37 °C. *A. hydrophila* was cultured in nutrient broth (Difco) at 30 °C overnight. *S. oneidensis* MR-1 was cultured in Tryptic soy broth (Difco) at 30 °C overnight.

*Saccharomyces cerevisiae* BY4742 (Euroscarf) was cultured in 1% yeast extract, 2% peptone and 2% glucose at 30 °C for 18 h. Spinach (*S. oleracea*) was purchased from a grocery store. *A. thaliana* Col-0 was purchased from Inplanta Innovations. Tobacco BY-2 cells (RIKEN BRC through the National BioResource Project) were cultured in modified Linsmaier and Skoog medium (pH 5.5), which contained Murashige and Skoog Plant Salt Mixture (Wako), 30 g l^−1^ sucrose, 0.2 g l^−1^ KH_2_PO_4_, 100 mg l^−1^ myo-inositol, 1 mg l^−1^ thiamine-HCl, 0.2 mg l^−1^ 2,4-dichlorophenoxyacetic acid and NaOH, in the dark at 25 °C with agitation at 130 rpm. The unicellular red alga *C. merolae* 10D was cultured in MA2 medium (pH 3)^47,48^ at 42 °C under LED light.

### Construction of plasmids and strains

The *V. cholerae Para-tilS* strain was created using homologous recombination and a derivative of the suicide vector pCVD442. The arabinose-inducible promoter with the *araC* gene (pBAD; Invitrogen) and ~1,000 base pairs of DNA flanking each side of the target were cloned into the SmaI site of pCVD442 using NEBuilder HiFi DNA Assembly Master Mix (New England Biolabs (NEB)). The endogenous *tilS* promoter was replaced with the arabinose-inducible promoter with *araC*.

*E. coli* and *V. cholerae* TilS protein-expressing vectors (pET28b-ECTilS and pET28b-VCTilS) were generated by integrating the *E. coli* or *V. cholerae TilS* open reading frame into linearized pET28 using NEBuilder HiFi DNA Assembly Master Mix (NEB).

Reporter protein-expressing plasmids for measuring Ile-decoding ability were generated by integrating tandem sequences testing decoding of the Ile codon into linearized pMMB207 encoding mCherry and bright GFP using NEBuilder HiFi DNA Assembly Master Mix (NEB). The DNA primers used to construct plasmids and strains are listed in Supplementary Table 4.

### RNA extraction

For *A. ferrooxidans*, budding yeast, *S. pombe*, *A. thaliana* and spinach cells were frozen with liquid nitrogen followed by homogenization with a prechilled mortar and pestle. The cell powder was suspended in a 1:1 mixture of water–saturated phenol and extraction buffer (50 mM NaOAc and 10 mM Mg(OAc)_2_, pH 5.2) and vigorously stirred for 1 h at room temperature. SDS and sarkosyl were added as needed. For *B. subtilis* and cyanobacteria, cells suspended in a 1:1 mixture of water–saturated phenol and extraction buffer were incubated at 95 °C for 20 min. For *E. coli*, *M. mobile*, *P. putida*, *G. kaustophilus*, *C. merolae*, *V. cholerae* and *H. marismortui* cells suspended in a 1:1 mixture of water–saturated phenol and extraction buffer were subjected to two freeze-thaw cycles using liquid N_2_, followed by vigorous stirring for 1 h at room temperature. The aqueous phase was separated by centrifugation and washed with chloroform. RNA was precipitated with 2-propanol. RNA dissolved in ultrapure water was cleaned up with TriPure (Roche) and precipitated with ethanol. The pellet was rinsed with 80% ethanol and dried. The obtained RNA was further separated by anion-exchange chromatography with DEAE Sepharose Fast Flow (Cytiva) or by polyacrylamide gel electrophoresis and gel slicing to enrich tRNAs. The tRNA mixture of *Thermus thermophilus* HB27 was kindly provided by N. Shigi (AIST).

### tRNA isolation

*V. cholerae* tRNA^Ile2^ was isolated by a batch-wise solid-phase DNA probe method^49^ from the total RNA fraction separated by anion-exchange chromatography. Typically, 2 mg of the RNA fraction was mixed with 200–400 μl of streptavidin agarose beads (Pierce) bound to 4 nmol of the biotinylated DNA probe (Supplementary Table 5) in 300 mM HEPES–KOH (pH 7.0), 1.2 M NaCl, 15 mM EDTA and 1 mM DTT at 68 °C for 30 min with shaking. The beads were washed three times with 15 mM HEPES–KOH (pH 7.0), 0.6 M NaCl, 7.5 mM EDTA and 1 mM DTT and seven times with 0.5 mM HEPES–KOH (pH 7.0), 20 mM NaCl, 0.25 mM EDTA and 1 mM DTT. Purified tRNAs were extracted from the beads with TRIzol (Thermo Fisher Scientific). After treating with Turbo DNase (Thermo Fisher Scientific) to remove residual DNA probes, tRNA was purified by 10% PAGE with 7 M urea.

tRNA sequences for isolation were obtained from PlantRNA^50^, tRNADB-CE^51^, tRNAdb^52^ and NCBI, compared and integrated (Supplementary Data 1). Chloroplast and/or mitochondrial tRNAs^Ile2^ from spinach, *A. thaliana* and *C. merolae* were homogeneously isolated by RCC using an automated RCC device, basically following the previously described protocol^31,32^. DNA probes complementary to each tRNA were designed using Raccess^53^ to have sufficient binding capability and specificity. The sequences of the DNA probes used in this study are listed in Supplementary Table 5. The 5′-EC amino-modified DNA probes (Sigma-Aldrich) were covalently immobilized on NHS-activated Sepharose 4 Fast Flow (Cytiva). The DNA resins packed in custom-made tips were set to a custom-made multichannel head on the RCC device. The tips were cleaned up with 50 mM NaOH before each RCC run. RNA dissolved in 6× NME buffer (1.2 M NaCl, 30 mM MES–NaOH (pH 6.0), 15 mM EDTA and 1 mM DTT) was passed through the tip by auto-pipetting at 65 °C. After washing the tip columns with 0.1× NME at 40 °C, bound tRNAs were eluted in 0.1× NME at 68 °C. Purity was confirmed by 10% PAGE with 7 M urea.

### Nucleoside analysis by MS

Four micrograms of total tRNA or ~10 pmol of isolated tRNA were digested with 0.05 U nuclease P_1_ (FUJIFILM Wako Pure Chemical) and 0.04 U bacterial alkaline phosphatase (BAP; from *E. coli* C75; Nippon Gene) in 20 mM NH_4_OAc (pH 5.3) at 37 °C for 1 h.

For normal-phase chromatography, hydrophilic interaction LC (HILIC)/ESI–MS was used for nucleoside analysis^54^. Nucleosides were dissolved in 90% acetonitrile/10% water and applied to a ZIC-cHILIC column (3 μm particle size, 2.1 × 150 mm; Merck Millipore) coupled with ESI–MS on a Q Exactive Hybrid Quadrupole-Orbitrap Mass Spectrometer (Thermo Fisher Scientific), equipped with an ESI source and an Ultimate 3000 LC system (Thermo Fisher Scientific). The mobile phase consisted of 5 mM NH_4_OAc (pH 5.3; solvent A) and acetonitrile (solvent B). The nucleosides were chromatographed with a flow rate of 100 μl min^−1^ in a multistep gradient as follows: linear 90–85% solvent B from 0 to 10 min, 85–30% solvent B from 10 to 30 min with curve 7, 30% solvent B for 10 min and then initialized to 90% solvent B. Proton adducts of nucleosides were scanned in a positive polarity mode over an *m*/*z* range of 103–700 or 900. Xcalibur 4.4 (Thermo Fisher Scientific) was used for system operation.

For reverse-phase chromatography/ESI–MS^55^, nucleosides were applied to a SunShell C18 column (2.6 μm particle size, 2.1 × 150 mm; ChromaNik Technologies) and analyzed by a Q Exactive system with the same solvents as described above. The nucleosides were chromatographed with a flow rate of 75 μl min^−1^ in a multistep gradient as follows: 0–15% solvent B from 0 to 30 min with curve 7, linear 15–60% solvent B from 30 to 35 min, 60% solvent B for 10 min and then initialized to 0% solvent B.

For Figs. 1f and 2a,b and Extended Data Figs. 5 and 6a, nucleosides of tRNAs or tRNA fractions were analyzed by dynamic multiple reaction monitoring (MRM) using Agilent 6460 QQQ (Agilent). One hundred nanograms of tRNA fraction or purified tRNA were digested with 0.5 U nuclease P1 (US Biological) and 0.1 U phosphodiesterase I (Sigma) in 22 μl reactions containing 50 mM Tris–HCl (pH 5.3) and 10 mM ZnCl_2_ at 37 °C for 1 h. Reaction mixtures were then mixed with 2 μl of 1 M Tris–HCl (pH 8.3) and 1 U μl^−1^ calf intestine phosphatase (Sigma) and incubated at 37 °C for 30 min. Enzymes were filtered out using 10K ultrafiltration columns (VWR). Then, 18 μl aliquots were mixed with 2 μl of 50 μM ^15^N-dA, and 2.5–10 μl digests were injected into an Agilent 1290 ultra-HPLC system bearing a Synergi Fusion-RP column (100 × 2 mm, 2.5 μm; Phenomenex) at 35 °C with a flow rate of 0.35 ml min^−1^ using a solvent system consisting of 5 mM NH_4_OAc (buffer A) and 100% acetonitrile (buffer B). The gradient of acetonitrile was as follows: 0%, 0–1 min; 0–10%, 1–10 min; 10–40%, 10–14 min; 40–80%, 14–15 min; 80–100%, 15–15.1 min; 100%, 15.1–18 min; 100–0%, 18–20 min and 0%, 20–26 min. The eluent was ionized by an ESI source and directly injected into the mass spectrometer with the following parameters: gas temperature, 250 °C; gas flow, 11 l min^−1^; nebulizer, 20 psi; sheath gas temperature, 300 °C; sheath gas flow, 12 l min^−1^; capillary voltage, 1,800 V and nozzle voltage, 2,000 V.

Dynamic MRM was carried out to survey known RNA modifications. The retention time windows and *m*/*z* values of precursor and product ions for dynamic MRM analyses are listed in Supplementary Data 2.

### RNA fragment analysis by MS

For RNA fragment analysis, 1 pmol of isolated tRNA was digested with 20 U RNase T_1_ (Thermo Fisher Scientific) in 20 mM NH_4_OAc (pH 5.3) at 37 °C for 1 h. The digests were mixed with a one-tenth volume of 0.1 M triethylamine acetate (pH 7.0) and subjected to LC-nano ESI–MS on an LTQ Orbitrap mass spectrometer (Thermo Fisher Scientific) equipped with a splitless nanoflow HPLC (nano-HPLC) system (DiNa; KYA Technologies) using a nano-LC trap column (C18, 0.1 × 0.5 mm; KYA Technologies) and a capillary column (HiQ Sil C18W-3, 0.1 × 100 mm; KYA Technologies)^32,55^. Digested fragments were separated for 35 min at a flow rate of 300 nl min^−1^ by capillary LC using a linear gradient from 2% to 100% solvent B in a solvent system consisting of 0.4 M 1,1,1,3,3,3-hexafluoro-2-propanol (HFIP; pH 7.0; solvent A) and 0.4 M HFIP (pH 7.0) in 50% methanol (solvent B). The eluent was ionized by an ESI source in a negative polarity mode and scanned over an *m*/*z* range of 600–2,000. Xcalibur 2.0.7 (Thermo Fisher Scientific) was used for system operation. The LC–MS data were analyzed using Qual Browser (Thermo Fisher Scientific). Excel was used to calculate the *m*/*z* value of each fragment.

### Purification of N^341^ nucleoside from spinach

Forty kilograms of fresh spinach were freeze-dried without blanching by Miyasaka Brewing Company and powdered by Mikasa Sangyo. Total RNA was extracted from 1.5 kg spinach powder with 15 l extraction buffer (50 mM NaOAc, 10 mM Mg(OAc)_2_, 0.2% SDS, 0.2% sarkosyl and 28.8 mM 2-mercaptoethanol (pH 5.2)) and 15 l water–saturated phenol by stirring with a mechanical stirrer for 3 h at room temperature. After centrifugation, the recovered upper phase was extracted using chloroform and subjected to 2-propanol precipitation. The RNA pellet was dissolved in ddH_2_O and purified by the AGPC method^56^. RNA was precipitated with 2-propanol, rinsed with 70% ethanol and dried. The obtained total RNA (total 14.4 g) was applied to a DEAE Sepharose FF column (Cytiva) equilibrated with buffer A (10 mM HEPES–KOH (pH 7.5) and 250 mM NaCl), washed with buffer A and then eluted with buffer B (10 mM HEPES–KOH (pH 7.5) and 1 M NaCl) to remove contaminants and long RNAs^57^.

Spinach chloroplast tRNA^Ile2^ was isolated from the obtained RNA by chaplet column chromatography^33^. In total, 400 nmol of 3′-EC-amino linker DNA probe (Supplementary Table 5) was immobilized on HiTrap NHS-activated HP columns (1 ml; Cytiva). In total, 3.26 mg tRNA^Ile2^ was isolated.

The isolated tRNA^Ile2^ was digested in 20 mM NH_4_OAc (pH 5.3) containing nuclease P_1_ (0.1 U per 40 μg tRNA) and BAP (0.15 U per 40 μg tRNA) at 37 °C. The solution was purified with a PoraPak Rxn RP column (Waters) to remove salts, enzymes and other pyrimidines. The column was washed with 5 mM NH_4_OAc (pH 5.3) buffer and eluted with 50% CH_3_CN. The eluates were dried and separated by reverse-phase HPLC with an HP1100 LC system (Agilent Technologies) equipped with an Inertsil ODS-3 column (5 μm, 10 mm × 250 mm; GL Sciences). The mobile phase consisted of 5 mM NH_4_OAc (pH 7.2; solvent A) and 60% acetonitrile (solvent B). The nucleosides were chromatographed with a flow rate of 1 ml min^−1^ in a multistep linear gradient as follows: 0–14% solvent B from 0 to 2 min, 14% solvent B for 15 min, 14–21% solvent B from 17 to 45 min, 21–99% solvent B from 45 to 55 min and then 99% solvent B for 20 min. The N^341^-rich fraction that was eluted around 50 min was collected (Supplementary Fig. 3a), dried and dissolved in 80% acetonitrile. The fraction was further purified using a ZIC HILIC column (5 μm, 10 mm × 150 mm; Merck Millipore) with a mobile phase of 5 mM NH_4_OAc (pH 5.3; solvent A) and acetonitrile (solvent B). N^341^ was separated with a flow rate of 0.2 ml min^−1^ in a multistep linear gradient as follows: 85–30% solvent B from 0 to 40 min, 30% solvent B for 10 min and then initialized to 85% B. N341 that eluted around 36 min was collected (Supplementary Fig. 3b), dried and desalted with a PoraPak Rxn RP column. Purity was confirmed by LC–MS analysis. The yield of N^341^ was about 0.16 A_260_ units.

### Deuterium exchange MS

One nanomole of purified N^341^ was dissolved in D_2_O (D, 99.9%; Cambridge Isotope Laboratories), incubated at 40 °C for 15 min and dried under a vacuum. This operation was repeated twice more. Deuterium-substituted N^341^ was dissolved in 50% acetonitrile/D_2_O (D, 99.96%; Cambridge Isotope Laboratories) and directly infused into an LTQ Orbitrap mass spectrometer (Thermo Fisher Scientific).

### Chemical synthesis of ava^2^C and NMR spectroscopy

The ava^2^C nucleoside was chemically synthesized by the scheme shown in Fig. 2f. 2-Thiocytidine was prepared as previously reported^58^. The other materials were purchased from commercial sources. 2-Thiocytidine (15.4 mg, 0.06 mmol) was mixed with sodium bicarbonate (4.4 mg, 0.052 mmol; FUJIFILM Wako Pure Chemical) and iodomethane (6.5 μl, 0.104 mmol; TCI) in 0.5 ml dried ethanol. The solution was incubated for 1 day to obtain 2-methylthiocytidine and centrifuged. The supernatant was collected and concentrated. To the residue, 5-AVA hydrochloride (7.9 mg, 0.052 mmol; Enamine) and sodium hydroxide (4.2 mg, 0.104 mmol; FUJIFILM Wako Pure Chemical) were added and then incubated in 1 ml dried ethanol for 3 days. Water and acetic acid were added to the final solution to adjust the pH and volume to 7 and 5 ml, respectively. The product was fractionated and purified by reverse-phase chromatography with an ODS column (COSMOSIL 5C_18_-MS-II; Nacalai Tesque; Extended Data Fig. 7a) using a gradient of triethylamine acetate buffer (0.2 M (pH 7.0); solvent A) to acetonitrile (0–10% solvent B from 0 to 20 min, 10–30% solvent B from 20 to 25 min and 30% solvent B from 25 to 30 min) to give 6.58 mg ava^2^C as a white powder (32% yield) after lyophilization. The product was analyzed by LC–MS (Fig. 2g and Supplementary Fig. 5) and NMR. ^1^H and COSY NMR spectra were measured with a JEOL JNM-ECS 400 instrument (Extended Data Fig. 7c–e). The chemical shifts are shown in parts per million using tetramethylsilane or solvent (DMSO-d_6_) as an internal standard. For ^1^H NMR (400 MHz, DMSO-d_6_; Extended Data Fig. 7c), the shifts are as follows: *δ* = 1.43–1.66, 2.07, 3.36 (11H, CH_2_ in AVA, 2′-OH, 3′-OH, 5′-OH), 3.54–3.75 (m, 2H, H5′), 3.92–4.13 (m, 3H, H4′, H3′, H2′), 5.71 (d, *J* = 5.3 Hz, 1H, H1′), 6.02 (d, *J* = 7.6 Hz, 1H, H5), 6.72 (s, 1H, 4-NH), 7.34 (s, 1H, 2-NH) and 8.02–8.32 (m, 3H, H6, CONH_2_ in AVA). For ^1^H NMR (400 MHz, DMSO-d_6_ + D_2_O; Extended Data Fig. 7d), the shifts are as follows: *δ* = 1.44–1.67, 2.11, 3.40 (8H, CH_2_ in AVA), 4.07 (ddd, *J* = 12.5, 5.2, 2.6 Hz, 2H, H4′, H3′), 4.18–4.24 (m, 1H, H2′), 5.62 (d, *J* = 6.3 Hz, 1H, H1′), 6.17 (d, *J* = 7.6 Hz, 1H, H5) and 8.07 (d, *J* = 7.7 Hz, 1H, H6). No exchangeable protons of amine (typically *δ* = 0.5–5) were observed. An exchangeable proton estimated to be at position *N*^4^ was observed. These findings indicate that the amino group of 5-AVA is bound to the C2 atom of the cytosine ring. LC–MS analysis, the calculated mass for the (M + H)⁺ ion of ava^2^C was 342.1777 (C_14_H_23_N_5_O_5_), and the observed mass was 342.1781.

### UV spectra of ava^2^C and L

The synthetic ava^2^C nucleoside was dissolved in 50 mM sodium phosphate buffer (pH 2, 3 and 6–9), sodium acetate (pH 4 and 5) or sodium borate (pH 10). UV spectra were measured with a BioDrop DUO+ instrument (Biochrom). The UV spectrum of L (NARD Institute) was also measured.

### Enzymatic reconstitution of ava^2^C, L, and t^6^A

The *E. coli* tRNA^Ile2^ transcript was synthesized by T7 run-off transcription^59,60^. Reconstitution of ava^2^C or L was carried out at 37 °C for 1 h in a reaction mixture containing 100 mM HEPES–KOH (pH 8.6), 50 mM KCl, 2 mM ATP, 2 mM DTT, 10 mM 5-AVA hydrochloride or l-lysine monohydrochloride (FUJIFILM Wako Pure Chemical), 1 μg transcribed tRNA and 1.5 μM *E. coli* TilS^60^. tRNAs were extracted with TriPure (Roche), precipitated twice with ethanol and rinsed twice with 80% ethanol.

Reconstitution of the t^6^A modification was carried out at 37 °C for 1.5 h in a reaction mixture containing 100 mM HEPES–KOH (pH 7.6), 25 mM MgCl_2_, 25 mM KCl, 5 mM DTT, 2 mM ATP, 10 mM NaHCO_3_, 5 mM l-threonine, 2.5 μM transcribed tRNA and 2.5 mM each *E. coli* TsaC, TsaD, TsaE and TsaB^60^. After the reaction, the tRNA was purified using a mixture of acidic phenol–chloroform–isoamyl alcohol (25:24:1), followed by NAP-5 gel filtration (Cytiva) and ethanol precipitation. The frequency of each modification introduced was monitored by LC–MS analysis.

For Supplementary Fig. 6, the in vitro reaction was conducted with *V. cholerae* tRNA^Ile2^ transcript, *E. coli* and *V. cholerae* TilS and a small compound fraction derived from *V. cholerae* cells. In a 20 μl reaction, 50 pmol tRNA^Ile2^ transcript was mixed with 1 μM *E. coli* or *V. cholerae* TilS, 1 mM ATP, 10 mM MgCl_2_ and 7 μl small compound fraction and incubated at 37 °C for 1 h. Reacted tRNAs were extracted using TRIzol (Thermo Fisher Scientific) and analyzed by LC–MS as described above.

### Aminoacylation assay

Isoleucylation of each tRNA was carried out at 37 °C in a reaction mixture consisting of 100 mM Tris–HCl (pH 7.8), 5 mM MgCl_2_, 10 mM KCl, 1 mM DTT, 2 mM ATP, 50 μM L-(U-^14^C) Ile (12.025 GBq mmol^−1^; Moravek Biochemicals), 0.4 μM tRNA and 1.68 μM recombinant *E. coli* IleRS. At different time points, an aliquot was spotted onto a Whatman 3MM filter, and radioactivity was measured by a liquid scintillation counter (PerkinElmer) as previously described^60^.

### Small compound fraction from *V. cholerae* cells

Five milliliters of a *V. cholerae* overnight culture were inoculated into 1 l LB medium and cultured at 37 °C until optical density (OD)_600_ reached 0.4. Cells were harvested by centrifugation and resuspended in 15 ml lysis buffer (300 mM NaCl, 10% glycerol, 50 mM Tris–HCl (pH 8.1) and 10 mM MgCl_2_) containing 6 U DNase I. Cells were disrupted using an Emulsiflex instrument (Avestin) and cleared by centrifugation. Then, 500 μl lysate was loaded onto a YM-10 Amicon filter (Merck Millipore) and spun at 4,000*g* for 30 min. The flowthrough fraction was collected and stored at −80 °C.

### Recombinant proteins

For *E. coli* and *V. cholerae* TilS, the BL21(DE3) strain transformed with pET28b encoding *E. coli tilS* or *V. cholerae tilS* was grown in 10 ml LB medium (50 μg ml^−1^ Km) overnight, inoculated into 1 l LB medium (50 μg ml^−1^ Km) and grown at 37 °C with shaking. When OD_600_ reached 0.3, the flask was chilled to 18 °C and shaken for 30 min. Protein expression was induced by the addition of 1 mM isopropyl β-d-1-thiogalactopyranoside (IPTG), and the flask was incubated with shaking at 18 °C for 24 h. Harvested cells were resuspended in 40 ml lysis buffer (50 mM Tris–HCl (pH 8.0), 10 mM MgCl_2_, 10% glycerol, 300 mM NaCl, 0.2 U ml^−1^ DNase I, 1 mM phenylmethylsulfonyl fluoride and complete proteinase inhibitor mixture (Roche)) and homogenized with an EmulsiFlex instrument (Avestin) for 20 min. The cleared lysate (35 ml) supplemented with 700 µl of 2 M imidazole (final concentration 40 mM) was mixed with 1.5 ml Ni-NTA beads equilibrated with 10 ml lysis buffer and incubated at 4 °C for 2.5 h with gentle rotation. Protein-bound beads were loaded on an open column (Bio-Rad) and washed twice with 10 m wash buffer (50 mM Tris–HCl (pH 8.0), 10 mM MgCl_2_, 10% glycerol, 300 mM NaCl and 40 mM imidazole). Protein was eluted with elution buffer 1 (50 mM Tris–HCl (pH 8.0), 10 mM MgCl_2_, 10% glycerol, 300 mM NaCl and 250 mM imidazole) and elution buffer 2 (50 mM Tris–HCl (pH 8.0), 10 mM MgCl_2_, 10% glycerol, 300 mM NaCl and 400 mM imidazole). The two elution fractions were mixed and dialyzed overnight in dialysis buffer 1 (20 mM Tris–HCl (pH 8.0), 300 mM NaCl, 10% glycerol and 1 mM DTT) and 8 h in dialysis buffer 2 (20 mM Tris–HCl (pH 8.0), 150 mM NaCl, 10% glycerol and 1 mM DTT). The protein concentration was measured by Qubit (Invitrogen).

### Reporter assay

The *V. cholerae Para-tilS* strain was transformed with the mCherry-GFP reporters harboring tandem Ile codons. Cells were cultured overnight in 2 ml LB medium (1 μg ml^−1^ Cm) at 30 °C and then diluted to OD_600_ = 0.01 in 5 ml LB medium containing 100 µM IPTG and 0.2% or 0.02% arabinose. Cells were cultured at 37 °C and harvested by centrifugation when OD_600_ reached 0.4. Harvested cells were resuspended in 100 μl PBS, mixed with 33 μl of 16% paraformaldehyde and incubated for 20 min at room temperature for fixation. Fixed cells were spun down, resuspended in 1 ml of PBS and left at room temperature overnight. The cell suspension was diluted 100-fold in 1 ml PBS and analyzed with a fluorescence-activated cell sorter (Sony, SH800S). GFP and mCherry signals were measured in 100,000 particles and analyzed with a custom R script. To decrease the background, particles with a signal intensity of less than 500 in the GFP or mCherry channel were excluded from the analysis. Relative log_2_(GFP/mCherry) values were used to evaluate the decoding activity of Ile codons.

### Metabolic labeling

*V. cholerae* cells were cultured in 500 μl of LB medium at 30 °C overnight and washed with 500 μl of M9 medium twice. Then, 5 μl of inoculum was mixed with M9 medium or M9 medium supplemented with full-label lysine (^13^C_6_, ^15^N_2_-lysine) or mono-labeled lysine (1-^13^C-lysine) and then cultured at 37 °C with shaking for 22 h. The tRNA fraction was enriched by removing long RNAs by precipitation with a low concentration of isopropanol^61^. Briefly, 250 μl of total RNA in 300 mM NaOAc (pH 5.5) was mixed with 200 μl of isopropanol, incubated at room temperature for 10 min and then centrifuged at 20,400*g* for 10 min at room temperature. The supernatant was collected, mixed with 50 μl of isopropanol and incubated at −20 °C for 30 min. A small RNA fraction was recovered by centrifugation. The enriched small RNA fraction was digested into nucleosides and analyzed by LC–MS as described above.

### Grid preparation and cryo-EM data collection

70S ribosomes were purified from *E. coli* MRE600 as previously described^27^. A series of synthetic mRNAs with or without 2′-OH substitutions at the fourth residue were purchased from Ajinomoto Bio-Pharma Service. The mRNA sequences are given in Supplementary Table 4. All mRNAs were gel purified by 10% PAGE with 7 M urea.

For complex formation, *E. coli* 70S ribosomes were mixed with mRNA and P-site tRNA in a solution containing 20 mM HEPES–KOH (pH 7.6), 10 mM Mg(OAc)_2_, 30 mM NH_4_Cl, 6 mM β-mercaptoethanol, 50 nM 70S ribosome, 500 nM mRNA and 500 nM P-site tRNA (*E. coli* tRNA-Glu or *P. putida* tRNA^Ile2^) at 37 °C for 30 min. Then, 500 nM of *P. putida* tRNA^Ile2^ was added and further incubated for 15 min. The resultant complexes were stabilized on ice for 30–60 min before grid preparation.

The grids were prepared using Vitrobot Mark IV (FEI) at 4 °C and 100% humidity. Quantifoil R1.2/1.3 300 mesh copper grids (Quantifoil) with a homemade thin carbon film were glow-discharged at 7 mA for 10 s with a PIB-10 Plasma Ion Bombarder (Vacuum Device). Thereafter, 3 µl of ribosome complex was applied to the grid, incubated for 30 s, blotted for 3 s with a blotting force of −10 and plunge-frozen in liquid ethane.

Automated data acquisition was performed using EPU 2.9 software (FEI) on a Krios G4 transmission electron microscope (FEI) operated at 300 kV. Images were acquired at the nominal magnification of ×105,000 with a defocus of 0.5–2.5 µm using a K3 direct electron detector (Gatan) in CDS-counting mode (0.8285 Å per pixel). The numbers of collected images for each ribosome complex are shown in Extended Data Fig. 9a. The collected images were fractionated into 48 frames with a total dose of 50 e^−^ Å^−^^2^.

### Image processing

Cryo-EM data processing was processed using RELION-3.1.2 (ref. ^62^). The movie frames were aligned with MotionCor2 (implemented with RELION), and CTF parameter estimation was performed with CTFFIND-4.1 (ref. ^63^). Particles were auto-picked using crYOLO^64^ with a box size of 530 pixels. The entire image processing procedure is summarized in Extended Data Fig. 9a.

For the ribosome complexed with A- and P-site *P. putida* tRNA^Ile2^, 801,672 particles were extracted from 6,134 images in a box size of 150 pixels (2.9274 Å per pixel). Then, 2D classification was performed, and subsets of 70S ribosomes were selected. A refined 3D map was used as a consensus map and subjected to 3D classification. Particles classified as well-resolved 70S ribosomes with sufficient P-site density were kept, and a 3D-refined volume was generated to perform A-site-focused 3D classification using a mask. Particles in the subsets with high occupancy at the A-site were selected, re-extracted in a box size of 530 pixels (without rescaling, 0.8285 Å per pixel) and subjected to 3D refinement followed by per-particle CTF refinement, Bayesian polishing and second 3D refinement. The generated map was sharpened by postprocessing, and the final resolution was 2.25 Å. Image processing for ribosome complexes with different mRNAs with 2′-OH substitution at the residue 3′-adjacent to the A-site codon was conducted using the same workflow as described above. The final resolutions of these complexes ranged from 2.39 to 2.47 Å.

### Model building

A starting model was assembled using published structures (Protein Data Bank (PDB) IDs: 7K00 (ref. ^65^) for 70S ribosome and 4V8N ref. ^41^ for A-site tRNA^Ile2^ and mRNA) and docked into the final map by Chimera^66^, followed by real-space refinement by Phenix^67^. The model of P-site *E. coli* tRNA^Glu^ was built by modifying the nucleotide sequence of tRNA^fMet^ (PDB ID: 7K00) with Coot^68^. The ligand restraints for tRNA modifications were generated by eLBOW implemented with Phenix.

### A-site binding assay

The A-site tRNA-binding assay was performed according to a previous study^11,60^ with modifications. For Fig. 3d, *E. coli* tRNA^Ile2^ transcripts bearing t^6^A37 and ava^2^C34 or L34 (50 pmol) were dephosphorylated in a 10 µl reaction mixture containing 0.05 U BAP and 10 mM HEPES–KOH (pH 7.6) at 55 °C for 30 min and gel purified. tRNA was 3′-labeled with (γ-^32^P)ATP (PerkinElmer) by T4 polynucleotide kinase (Toyobo) according to the manufacturer’s instructions and gel purified. Radioactivity was quantified by Cherenkov counting. mRNA containing the AUA or AUG codon was synthesized by T7 run-off transcription^59^. The mRNA sequences are given in Supplementary Table 4. The P-site of the *E. coli* 70S ribosome was occupied with native *E. coli* tRNA^Glu^. A 10 µl mixture containing 2.5 pmol 70S ribosome, 20 pmol *E. coli* tRNA^Glu^ and 25 pmol mRNA in binding buffer (50 mM HEPES–KOH (pH 7.6), 60 mM KCl, 6.5 mM Mg(OAc)_2_, 1 mM DTT and 0.5 mM spermine) was incubated at 37 °C for 30 min. Then, the tRNA solution (10 µl) containing 2.5 pmol 3′-^32^P-labeled tRNA (20,000 cpm) in binding buffer was added to the mixture, followed by further incubation at 37 °C for 15 min. The same mixture without mRNA was used as a negative control. The mixture (20 µl) was dot-blotted onto double-layered nitrocellulose (Protran Premium; Cytiva) and nylon (Hybond-N+; Cytiva) membranes and washed twice with 200 µl binding buffer. The membranes were exposed to an imaging plate, and radioactivity on the spots was visualized by a FLA-7000 image analyzer (Fujifilm) and quantified with Multi Gauge V3.0 (Fujifilm).

For Fig. 5b, a series of synthetic mRNAs with different 2′-OH substitutions at the residue 3′-adjacent to the A-site codon were purchased from Ajinomoto Bio-Pharma Service. The mRNA sequences are given in Supplementary Table 4. All mRNAs were gel purified by 10% PAGE with 7 M urea. *P. putida* tRNA^Ile2^ was 3′-labeled with (γ-^32^P) ATP and T4 polynucleotide kinase (Toyobo). First, the P-site of the *E. coli* 70S ribosome was occupied with *E. coli* tRNA^Glu^ at 37 °C for 30 min in a mixture (5 µl) containing 0.3 pmol *E. coli* 70S ribosome, 3 pmol *E. coli* tRNA^Glu^ and 10 pmol mRNA in binding buffer (50 mM HEPES–KOH (pH 7.6), 120 mM KCl, 6.5 mM Mg(OAc)_2_, 1 mM DTT and 0.5 mM spermine). Then, the tRNA solution (5 µl) containing 0.5 pmol 3′-^32^P-labeled tRNA (5,000 cpm) in binding buffer was added to the mixture, followed by further incubation at 37 °C for 30 min. No mRNA was used as a negative control. The mixture (10 µl) was added to 60 µl binding buffer, dot-blotted onto double-layered nitrocellulose (GE Healthcare) and nylon (Hybond-N+; GE Healthcare) membranes and then washed three times with 200 µl binding buffer. The membranes were exposed to an imaging plate, and radioactivity on the spots was visualized by a FLA-7000 image analyzer (Fujifilm) and quantified with Multi Gauge V3.0 (Fujifilm). The binding ratio was calculated, and the statistical test was performed using Microsoft Excel.

### Figure preparation

All figures were prepared with Canvas X (Nihon Poladigital K.K.) using the outputs of other softwares. Chemical structures were drawn with ChemDraw (PerkinElmer). The density maps and atomic models in Figs. 4 and 5 and Extended Data Figs. 9 and 10 were generated with Chimera and ChimeraX^69^. Bar graphs were generated using GraphPad Prism 7.04, 8 and 9.3.1 (GraphPad Software).

### Reporting summary

Further information on research design is available in the Nature Portfolio Reporting Summary linked to this article.

## Online content

Any methods, additional references, Nature Portfolio reporting summaries, source data, extended data, supplementary information, acknowledgements, peer review information; details of author contributions and competing interests; and statements of data and code availability are available at 10.1038/s41589-024-01726-x.

## Supplementary information

Supplementary Information Supplementary Figs. 1–7 and Supplementary Tables 1–5.

Reporting Summary

Supplementary Data 1 tRNA species and their sequences in AUN codon box.

Supplementary Data 2 Retention time windows and *m*/*z* values of precursor and product ions for dynamic MRM.

## Source data

Source Data Fig. 3 Statistical source data.

Source Data Fig. 5 Statistical source data.

Source Data Extended Data Fig. 4 Unprocessed gels.

Source Data Extended Data Fig. 5 Statistical source data.

Source Data Extended Data Fig. 7 Statistical source data.

Source Data Extended Data Fig. 8 Unprocessed gels.

## Data Availability

Publicly available datasets from the PDB (7K00, 4V8N and 4V5R) were used for atomic model building and comparison. Cryo-EM maps and atomic coordinates of the reported structures were deposited in Electron Microscopy Data Bank and PDB, respectively, with the following accession codes: EMD-39577 and 8YUO (U4 mRNA); EMD-39578 and 8YUP (A4 mRNA); EMD-39579 and 8YUQ (dA4 mRNA); EMD-39580 and 8YUR (Am4 mRNA); and EMD-39581 and 8YUS (A(F)4 mRNA). See Supplementary Data 1 for the databases and accession codes referred to for obtaining tRNA sequences. Source data are provided with this paper.
