# Supplementary material for: A tRNA modification with aminovaleramide facilitates AUA decoding in protein synthesis
Source: Nat Chem Biol. 2024 Sep 19;21(4):522–31. doi: 10.1038/s41589-024-01726-x (PMC11938285; doi:10.1038/s41589-024-01726-x)
Supplement: Supplementary file 1 — Supplementary Figs. 1–7 and Supplementary Tables 1–5. [file 41589_2024_1726_MOESM1_ESM.pdf]

nature chemical biology

Supplementary information

https://doi.org/10.1038/s41589-024-01726-x

A tRNA modification with aminovaleramide facilitates AUA decoding in protein synthesis

In the format provided by the authors and unedited

Table of contents

Supplementary Fig. 1. Isolation of $tRNAs^{Ile2}$ from plant organelles.
Supplementary Fig. 2. Phylogenetic distribution of N341 and L in domains of life.
Supplementary Fig. 3. Large-scale purification of N341 from spinach.
Supplementary Fig. 4. Enzymatic synthesis of ava^2C34 by *E. coli* TilS.
Supplementary Fig. 5. Comparison of CID spectra of native N341 and synthetic ava^2C.
Supplementary Fig. 6. *V. cholerae* TilS synthesizes L, not ava^2C, in the presence of its own metabolite fraction.
Supplementary Fig. 7. Source data for Supplementary Fig. 1 and 6.
Supplementary Table 1. Assignment of RNA fragments of isolated tRNAs.
Supplementary Table 2. Cryo-EM data collection, refinement and validation statistics
Supplementary Table 3. List of organisms used in this study
Supplementary Table 4. List of DNA oligos and mRNAs used in this study
Supplementary Table 5. Sequences of probes for tRNA isolation

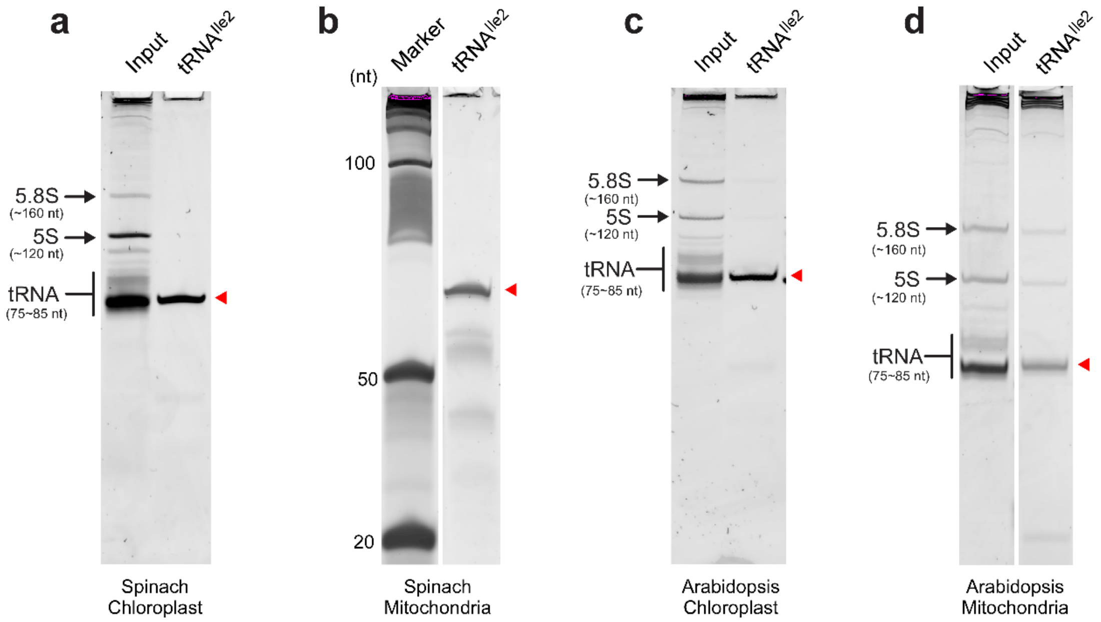

Supplementary Fig. 1. Isolation of tRNAs^{Ile2} from plant organelles

Gel analyses of purified tRNA^{Ile2} from spinach chloroplasts (a) and mitochondria (b), and *A. thaliana* chloroplasts (c) and mitochondria (d). Input fractions, RNA marker (DynaMarker RNA Low II, BioDynamics Laboratory), and purified tRNA^{Ile2} (indicated by red arrowhead) were resolved by 10% PAGE with 7 M urea. The RNAs were stained with SYBR Gold and visualized by a FLA-7000 scanner (Fujifilm). Source data are provided in Supplementary Fig. 7.

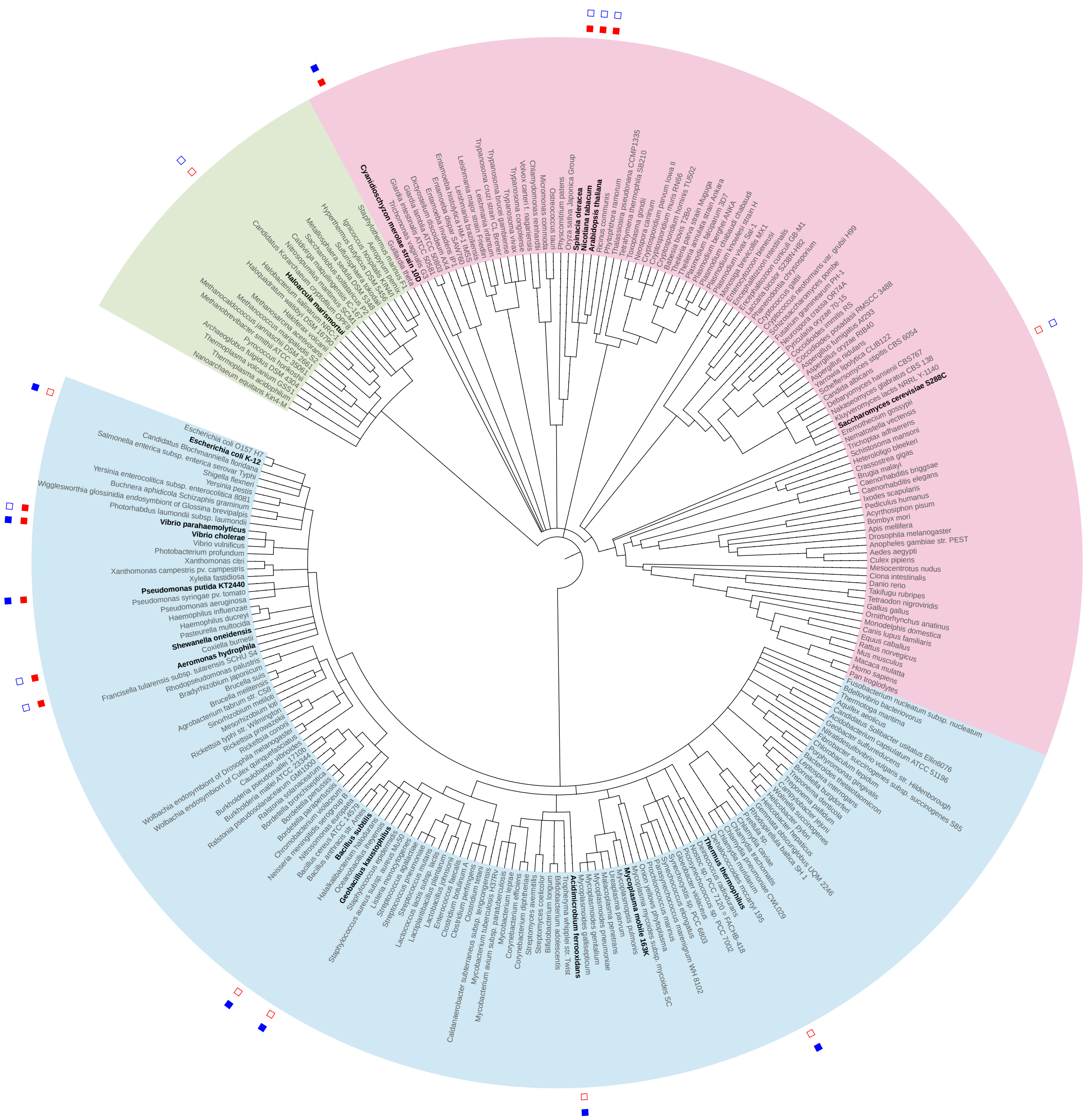

Supplementary Fig. 2. Phylogenetic distribution of N341 and L in domains of life. Phylogenetic distribution of N341 (red squares) and L (blue squares) in tRNA-rich fractions obtained from bacteria (pale blue), eukaryotes (pink), and archaea (pale green). The presence or absence of the modification (N341 or L) is shown by filled or unfilled squares, respectively. A phylogenetic tree of the organisms was constructed based on the NCBI taxonomy using phyloT v2 server (https://phylot.biobyte.de) and drawn by the iTOL v6 server (https://itol.embl.de).

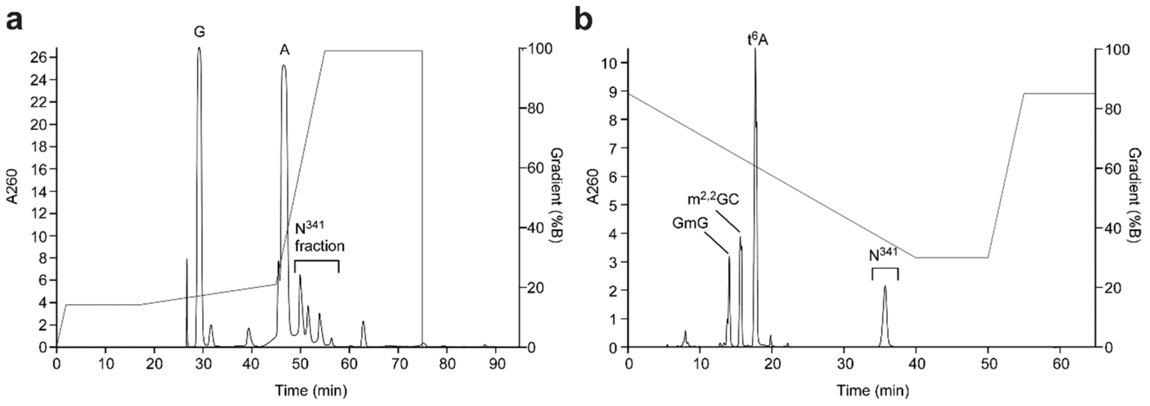

Supplementary Fig. 3. Large-scale purification of N341 from spinach.

a. Isolated spinach chloroplast tRNA^{Ile2} was digested by nuclease P1 and BAP, followed by rough purification by Porapak (Waters). The obtained purine-rich fraction was fractionated by HPLC with an Inertsil ODS-3 column (GL Science).

b. The N^{341}-rich fraction obtained in **a** was further purified by HILIC to obtain N^{341} nucleoside.

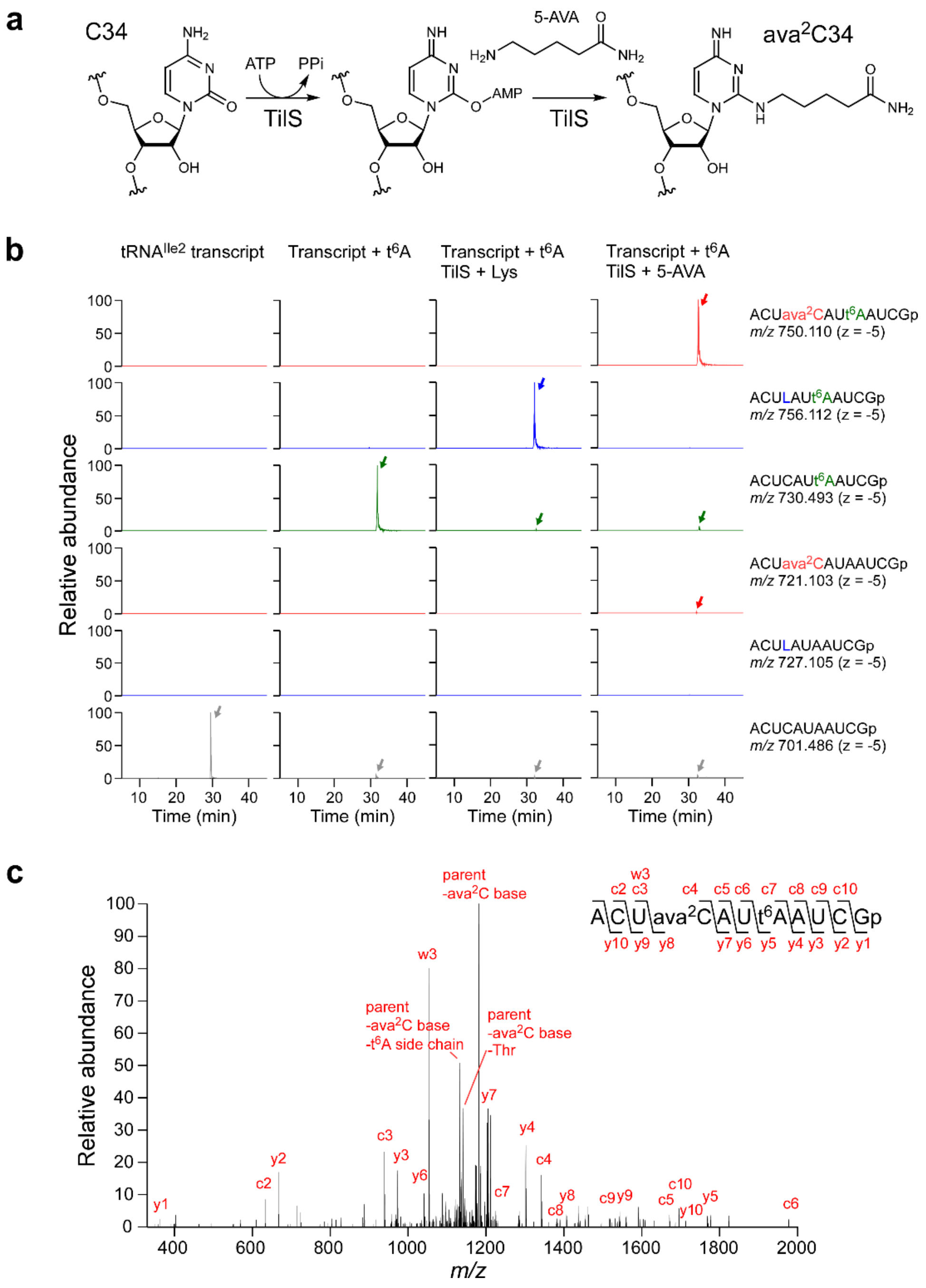

Supplementary Fig. 4. Enzymatic synthesis of ava²C34 by *E. coli* TilS.

a. Reaction scheme of the enzymatic synthesis of ava²C by TilS and 5-AVA.

b. LC-MS analyses of the tRNA modifications reconstituted *in vitro*. XICs of the modified tRNA^{Ile2} transcripts digested by RNase T_1 to detect different modification statuses. t⁶A37, L34, and ava²C34 were efficiently introduced at high frequencies.

c. CID spectrum of the RNA fragment containing both ava²C34 and t⁶A37. Product ions are assigned to the expected RNA sequence.

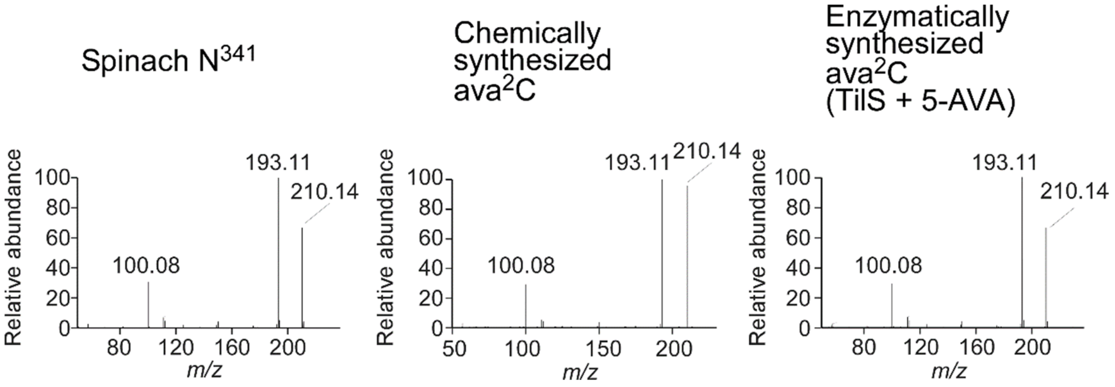

Supplementary Fig. 5. Comparison of CID spectra of native N^{341} and synthetic ava^2C. CID spectra of native N^{341} (left) and chemically (middle) and enzymatically (right) synthesized ava^2C nucleosides. The patterns of these CID spectra are almost identical. Interpretation of the fragmentation pattern is shown in **Fig. 2d**.

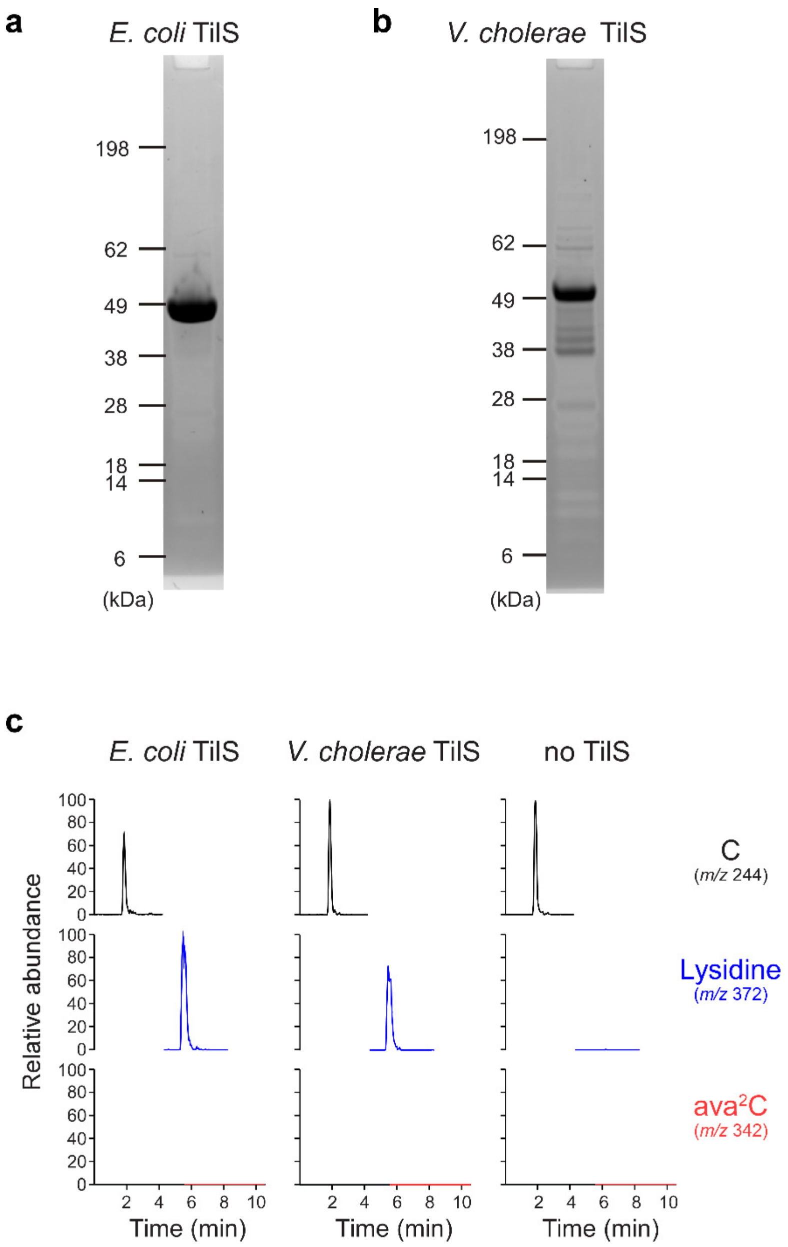

Supplementary Fig. 6. *V. cholerae* TilS synthesizes L, not ava^{2}C, in the presence of its own metabolite fraction.

a, b. Gel image of purified recombinant *E. coli* TilS (**a**) and *V. cholerae* TilS (**b**). 4-12% NuPAGE gels were stained with Coomassie Brilliant blue. The size of markers is indicated. Source data are provided in Supplementary Fig. 7.

c. LC-MS nucleoside analyses of tRNA^{Ile2} transcripts incubated with *E. coli* (left panels) or *V. cholerae* (middle panels) TilS in the presence of a small compound fraction (metabolites) extracted from *V. cholerae*. L was detected, but ava^{2}C was not.

Supplementary Figure 7 Unprocessed gel images

For Supplemetary Figure 1

a spinach chloroplast **b** Spinach mitochondria **c** Arabidopsis chloroplast **d** Arabidopsis mitochondria

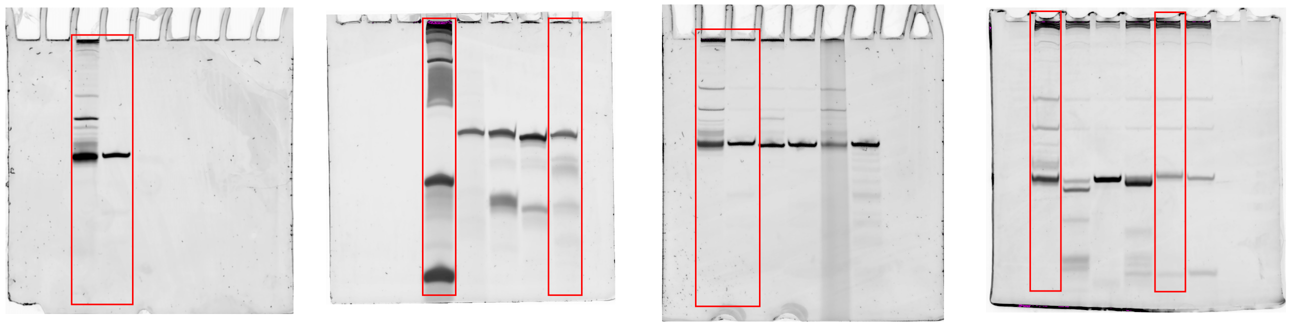

For Supplemetary Figure 6 Quality of recombinant proteins

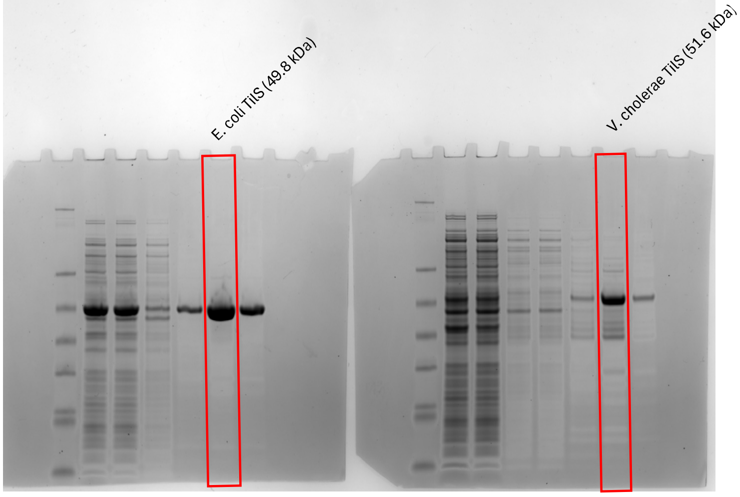

Supplementary Table 1. Assignment of RNA fragments of isolated tRNAs.

a) *S. oleracea* chroloplast tRNA^{Ile2} digested by RNase A

Sequences of RNase A fragments	Observed *m/z*	Theoretical *m/z*	charge state (z)	Difference (ppm)
AAAGCp	1654.248	1654.249	-1	0.60
AGGm^{5}Up	1356.193	1356.191	-1	1.47
GGAUp	1342.176	1342.175	-1	0.75
GAAUp	1326.182	1326.181	-1	0.75
t^{6}AAΨp	1126.171	1126.171	-1	0
N^{341}AUp	1055.207	1055.206	-1	0.95
GmGDp	1029.154	1029.154	-1	0
GGCp	1012.139	1012.139	-1	0
AAUp	981.136	981.133	-1	3.06
AACp	980.150	980.149	-1	1.02
pGCp	747.057	747.058	-1	1.34
m^{2,2}GCp	695.122	695.123	-1	1.44
GUp	668.076	668.075	-1	1.5
GCp	667.091	667.091	-1	0
AUp	652.082	652.081	-1	1.53
ACp	651.097	651.097	-1	0

b) *S. oleracea* chroloplast tRNA^{Ile2} digested by RNase T$_1$

Sequences of RNase T$_1$ fragments	Observed *m/z*	Theoretical *m/z*	charge state (z)	Difference (ppm)
Cm^{2,2}GCCCAACUN^{341}AUt^{6}AAΨΨGp	810.260	810.259	-7	1.23
CCCAACUN^{341}AUt^{6}AAΨΨGp	713.387	713.385	-7	2.80
m^{5}UΨCAAUUCCUACUGp	630.502	630.499	-7	4.76
CAUCCAUGp	636.078	636.077	-4	1.57
DDAAAGp	982.141	982.141	-2	0
AAUUCGp	968.120	968.120	-2	0
AAUGmGp	842.118	842.118	-2	0
CACCA	754.629	754.629	-2	0
AUGp	997.128	997.128	-1	0
UAGp	997.128	997.128	-1	0
CUGp	973.117	973.117	-1	0
CGp	667.092	667.091	-1	1.5

c) *S. oleracea* mitochondrial tRNA^{Ile2} digested by RNase T$_1$

Sequences of RNase T$_1$ fragments	Observed *m/z*	Theoretical *m/z*	charge state (z)	Difference (ppm)
AAACm^{2,2}GUACCGp	1635.744	1635.74	-2	2.35
CCCUACUAAGp	1590.21	1590.208	-2	1.37
CUN^{341}AUAACGp	1486.728	1486.729	-2	0.93
UUUAADDGmGp	1456.186	1456.184	-2	1.28
UUAUAUUGp	1274.637	1274.637	-2	0.04
CUUAUAGp	1121.132	1121.132	-2	0.26
CCUACCA	1060.161	1060.162	-2	0.98
UACCGp	1607.212	1607.211	-1	0.89
m^{5}UUCGp	1293.157	1293.158	-1	0.55
UAGp	997.126	997.128	-1	2
DDGp	978.132	978.132	-1	0.08
AGp	691.103	691.103	-1	0.44

d) *C. merolae* chroloplast tRNA^{Ile2} digested by RNase T$_1$

Sequences of RNase T$_1$ fragments	Observed *m/z*	Theoretical *m/z*	charge state (z)	Difference (ppm)
ACULAUt^{6}AAUCCGp	1362.535	1362.539	-3	3.44
ACUN^{341}AUt^{6}AAUCCGp	1352.534	1352.536	-3	1.61
ACUCAUt^{6}AAUCCGp	1319.833	1319.841	-3	5.89
CAUCUAUGp	1273.649	1273.653	-2	3.06
AAUCCAGp	1132.15	1132.154	-2	3.05
CDUAAGp	969.127	969.127	-2	0.81
AAUUUGp	968.61	968.612	-2	1.19
UCAUCGp	956.113	956.114	-2	1.5
ACAAGp	1654.244	1654.249	-1	3.3
CACCA	1510.261	1510.265	-1	3.18
m^{5}UUCGp	1293.155	1293.158	-1	2.35
AUGp	997.127	997.128	-1	1.05
CAGp	996.1	996.1	-1	3.39
CUGp	973.1	973.1	-1	0.99
CCGp	972.1	972.1	-1	0.34
AGp	691.1	691.1	-1	2.05
UGp	668.1	668.1	-1	1.82
CGp	667.1	667.1	-1	1.66

e) *P. putida* tRNA^{Ile2} digested by RNase T$_1$

Sequences of RNase T1 fragments	Observed *m/z*	Theoretical *m/z*	charge state (z)	Difference (ppm)
ACUava^{2}CAUct^{6}AAUCCUUUGp / ACUava^{2}CAUct^{6}AAΨCCUUUGp	1652.557	1652.558	-3	0.61
ACUava^{2}CAUt^{6}AAUCCUUUGp / ACUava^{2}CAUt^{6}AAΨCCUUUGp	1658.56	1658.561	-3	0.60
m^{5}UUCAAGp/m^{5}UΨCAAGp	975.128	975.127	-2	1.03
m^{7}Gacp^{3}UCCACGp	790.116	790.116	-3	0.00
CCUAUAGp	1120.64	1120.64	-2	0.00
CCCACCA	1059.67	1059.67	-2	0.00
CUCAGp	803.103	803.101	-2	2.49
DDAGp	653.09	653.088	-2	3.06
UCCGp	638.577	638.575	-2	3.13
CAGp	996.143	996.144	-1	1.00
DDGp	978.134	978.132	-1	2.04
AGp	691.105	691.103	-1	2.89
UGp	668.078	668.075	-1	4.49

Supplementary Table 2. Cryo-EM data collection, refinement and validation statistics

	#1<br>70S ribosome with A-, P-site PptRNA^{Ile2}<br>(EMD-39577)<br>(PDB 8YUO)	#2<br>70S ribosome with A-site PptRNA^{Ile2} on A4 mRNA<br>(EMD-39578)<br>(PDB 8YUP)	#3<br>70S ribosome with A-site PptRNA^{Ile2} on dA4 mRNA<br>(EMD-39579)<br>(PDB 8YUQ)	#4<br>70S ribosome with A-site PptRNA^{Ile2} on Am4 mRNA<br>(EMD-39580)<br>(PDB 8YUR)	#5<br>70S ribosome with A-site PptRNA^{Ile2} on A(F)4 mRNA<br>(EMD-39581)<br>(PDB 8YUS)
**Data collection and processing**					
Magnification	105000	105000	105000	105000	105000
Voltage (kV)	300	300	300	300	300
Electron exposure (e–/Å^2)	50	50	50	50	50
Defocus range (μm)	0.5-2.5	0.5-2.5	0.5-2.5	0.5-2.5	0.5-2.5
Pixel size (Å)	0.8285	0.8285	0.8285	0.8285	0.8285
Symmetry imposed	C1	C1	C1	C1	C1
Initial particle images (no.)	801672	554938	954339	747292	887279
Final particle images (no.)	339441	156265	239915	195411	281544
Map resolution (Å)	2.25	2.39	2.41	2.47	2.43
FSC threshold	0.143	0.143	0.143	0.143	0.143
Map resolution range (Å)					
**Refinement**					
Initial model used (PDB code)	7k00	7k00	7k00	7k00	7k00
Model resolution (Å)	2.25	2.39	2.41	2.47	2.43
FSC threshold	0.5	0.5	0.5	0.5	0.5
Model resolution range (Å)					
Map sharpening *B* factor (Å^2)	-41.1125	-42.601	-45.4352	-47.1922	-46.5162
Model composition					
Non-hydrogen atoms	141926	141910	141909	141911	141910
Protein residues	5587	5587	5587	5587	5587
RNA residues	4556	4556	4556	4556	4556
Ligands	309	309	309	309	309
B factors (Å^2)					
Protein	0	0	0	0	0
RNA	0	0	0	0	0
Ligand	0	0	0	0	0
R.m.s. deviations					
Bond lengths (Å)	0.004	0.003	0.006	0.004	0.006
Bond angles (°)	0.684	0.613	0.837	0.719	0.802
Validation					
MolProbity score	1.65	1.61	1.71	1.71	1.68
Clashscore	6.35	6.28	6.78	7.14	6.79
Poor rotamers (%)	0	0	0	0	0.07
Ramachandran plot					
Favored (%)	95.65	96.15	95.1	95.45	95.65
Allowed (%)	4.29	3.78	4.82	4.47	4.29
Disallowed (%)	0.05	0.07	0.07	0.07	0.05

Supplementary Table 3. List of organisms used in this study

Organism	Strain	Relevant genotype/description	Reference/source
Escherichia coli	MG1655	wild-type MG1655	
Escherichia coli	DH5α λpir	Cloning strain	
Escherichia coli	SM10 λpir	Conjugation strain	
Escherichia coli	BW25113		NBRP-E.coli at National Institute of Genetics, Japan
Vibrio cholerae	C6706	wild-type ElTor clinical isolate (SmR)	
Vibrio cholerae	C6706 *Para-tilS*		This study
Vibrio parahaemolyticus	RIMD 2210633	clinical isolate of the pandemic O3:K6 serotype	
Aeromonas hydrophila	ATCC7966		ATCC(American Type Culture Collection), USA
Shewanell oneidensis	MR-1		ATCC(American Type Culture Collection), USA
Pseudomonas putida	NBRC 14164		NBRC (NITE Biological Resource Center), Japan
Bacillus subtilis subsp. *subtilis*	str. 168		Akiko Soma (Chiba University)
Acidithiobacillus ferrooxidans	DSM 10331		NBRC (NITE Biological Resource Center), Japan
Geobacillus kaustophilus	NBRC 102445		NBRC (NITE Biological Resource Center), Japan
Thermus thermophilus	HB27		Naoki Shigi (AIST, Japan)
Mycoplasma mobile			Makoto Miyata (Osaka City University)
Haloarcula marismortui			Taketomo Fujiwara (Shizuoka University)
Saccharomyces cerevisiae	BY4742		Euroscarf
Cyanidioschyzon merolae	10D		Akiko Soma (Chiba University)
Spinacia oleracea			(Grocery store)
Arabidopsis thaliana	Col-0		Inplanta Innovations Inc., Japan
Nicotiana tabacum	BY-2		RIKEN BRC through the National BioResource Project, Japan

Supplementary Table 4. List of DNA oligos and mRNAs used in this study

Aim	DNA oligo name	5' -> 3'
Amplifying AraC and Para	pBAD33_Para_f	atcgatgcataatgtgcctgt
	pBAD33_Para_r	ttcgctaaagggctagccca
Amplifying 5' flanking region of *V. cholerae tilS*	VC2242_Para_5end_f	CCGCATGCGATATCGAGCTCTCCCattcaggcgctgcgtga
	VC2242_Para_5end_r	TTTGACAGGCACATTATGCATCGATtaagccaaccctttctatatgtgg
Amplifying 3' flanking region of *V. cholerae tilS*	VC2242_Para_3end_f	TTTTTTGGGCTAGCCCTTTAGCGAActtttctcaactctctgtggttta
	VC2242_Para_3end_r	CGGATAACAATTTGTGGAATTCCCactaatgcactgcgccag
Cloning *V. cholerae tilS* to pET28	VC2242_pET28_f	ACTTTAAGAAGGAGATATACCATGgacgatctctatctccactttgtg
	VC2242_pET28_r	CTCGAGTGCGGCCGCAAGCTTGTCttttggcacagaatcatgagattt
Cloning *E. coli tilS* to pET28	ECTilS_pET28_f	ACTTTAAGAAGGAGATATACCATGacactcacgctcaatagaca
	ECTilS_pET28_r	CTCGAGTGCGGCCGCAAGCTTGTCactaagcgttttctgccaga
DNA template for *in vitro* transcription of tRNA-Ile2	T7_VCtRNAIle2_f	GCTAATACGACTCACTATAGGCCCCTTAGCTCAGTGGTTAGAGCAGGCGACTCATAATCG
	T7_VCtRNAIle2_r	TGGTGGCCCCTCCCAGGTTCGAACTGGGGACCAAGCGATTATGAGTCGCCTGCTC
Linearizing pET28 for cloning	pET28_linear_f	GACAAGCTTGCGGCCGCA
	pET28_linear_r	CATGGTATATCTCCTTCTTAAAGTTAAACAA
Linearizing pMMB207-bGFP	pbGFP_linearize_f	ATGGCTAGCAAAGGAGAAGAACTT
	pbGFP_linearize_r	TTTAATAAACCTCCTTTCgggtaccga
Making AUC reporter construct	bGFP_Ile1_r	TCCTTTGCTgatgatttcgatgatAGCCATTTTAATAAACCTCCTTTC
	bGFP_Ile1_f	atcatcgaaatcatcAGCAAAGGAGAAGAACTTTTCACTGGAGTTGTC
Making AUC reporter construct	bGFP_Ile2_r	TCCTTTGCTtattatttctattatAGCCATTTTAATAAACCTCCTTTC
	bGFP_Ile2_f	ataatagaaataataAGCAAAGGAGAAGAACTTTTCACTGGAGTTGTC
Inserting linker and SD sequence between mCherry and bGFP	Separate-mCherry-bGFP-f_rev	AAGTTCTTCTCCTTTGCTAGCCATTTTAATAAACCTCCTTTCgggtac
	Separate-mCherry-bGFP-r_rev	GAGCTGTACAAGTCGCGAtgaagtacccGAAAGGAGGTTTAT
Amplifying a mCherry insert to pMMB207-bGFP	mCherry_f	gtacccGAAAGGAGGTTTATTAAAATGGTGAGCAAGGGCGAG
	mCherry_r	AAGTTCTTCTCCTTTGCTAGCCATTCGCGACTTGTACAGCTCG

Aim	mRNA name	5' -> 3' (*Italic* : P-site codon, **Bold**: A-site codon, Underlined: substitution)
A-site tRNA binding assay for Fig, 3d	AUA codon	5’-GGGUUAACUUAAGUAAGGAGGUAUACU*GAG* **AUA**UAACUGCAGAAAAAA-3’
	AUG codon	5’-GGGUUAACUUAAGUAAGGAGGUAUACU*GAG* **AUG**UAACUGCAGAAAAAA-3’
A-site tRNA binding assay for Fig, 5b and cryo-EM analyses	A-, P-site AUA codon	5'-GGGUUAACUUAAGUAAGGAGGUUUACU*AUA* **AUA**UAACUGCAGAAAAAA-3'
	A4 (2’-OH)	5’- GGUAAGGAGGUUUACU*GAG* **AUA**AAACUGCAAAAAA-3’
	dA4 (2’-H)	5’- GGUAAGGAGGUUUACU*GAG* **AUA**(dA)AACUGCAAAAAA-3’
	Am4 (2’-OCH_3)	5’- GGUAAGGAGGUUUACU*GAG* **AUA**(Am)AACUGCAAAAAA-3’
	A(F)4 (2’-F)	5’- GGUAAGGAGGUUUACU*GAG* **AUA**(A(F))AACUGCAAAAAA-3’

Supplementary Table 5. Sequences of probes for tRNA isolation

Organism	Organelle	tRNA	anticcodon	probe seq	length
Spinacia oleracea	chloroplast	Ile2	CAU	TGAGTTGGGCGCTTTAACCATTCAGCCATGGATGC	35
	mitochondria	Ile	CAU	TGGTAGGCTTAGTAGGGCTCGAACCTACAATATAA	35
Arabidopsis thaliana	chloroplast	Ile2	CAU	TGAGTTGGGCGCTTTAACCATTCAGCCATGGATGC	35
	mitochondria	Ile	CAU	TGGTAGGCTTAGTAGGGCTCGAACCTACAATATAA	35
Cyanidioschyzon merolae strain 10D	chloroplast	Ile2	CAU	AGTCCGCTGCCTTAAGCCGCTCGGCCATAGATGCA	35
E. coli		Ile2	CAU	TAAGTCGCCTGCTCTAACCACTGAGCTAAAGGGCC	35
Pseudomonas putida		Ile2	CAU	GAGTCCTCTGCTCTAACCAACTGAGCTATAGGCCC	35
Vibrio cholerae		Ile2	CAU	TGGCCCCTCCCAGGTTCGAACTGGGGACCAAGCGA	35
