# Supplementary figures and images for: A tRNA modification with aminovaleramide facilitates AUA decoding in protein synthesis
Source: Nat Chem Biol. 2024 Sep 19;21(4):522–31. doi: 10.1038/s41589-024-01726-x (PMC11938285; doi:10.1038/s41589-024-01726-x)

Gel images  
Extended Data Fig. 4

**b** Schyzon chloroplast

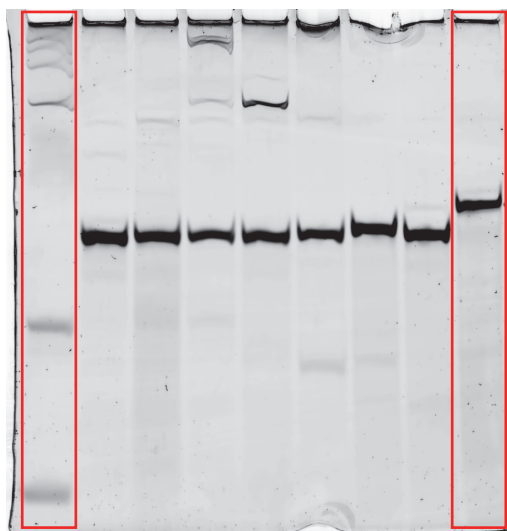

Supplement: Supplementary file 7 — Unprocessed gels. [file 41589_2024_1726_MOESM7_ESM.pdf]

Gel image

Extended Data Fig. 8b

*P. putida* tRNA<sup>Ile2</sup>

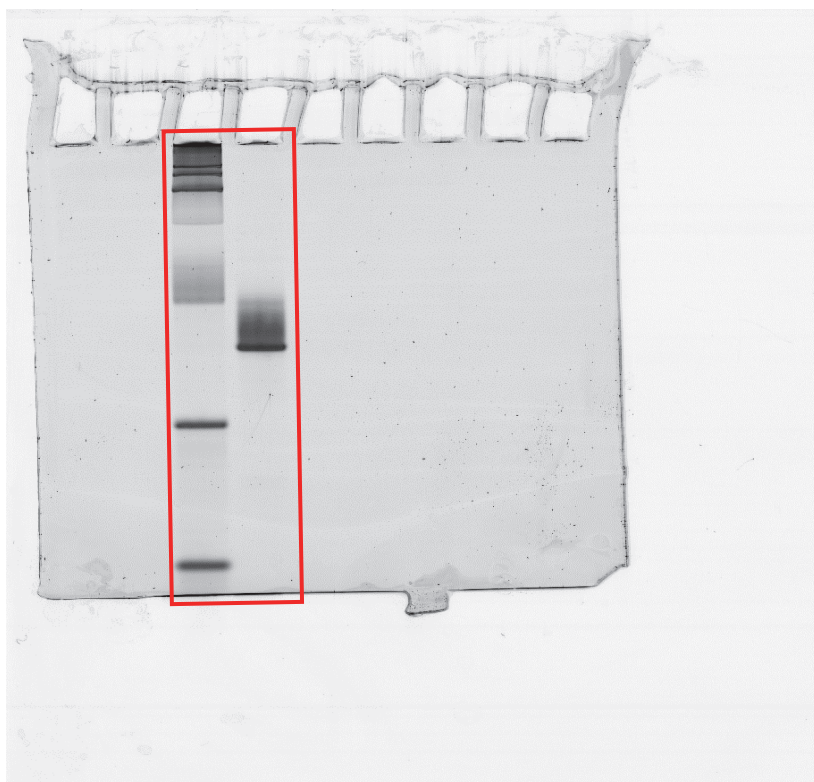

Supplement: Supplementary file 10 — Unprocessed gels. [file 41589_2024_1726_MOESM10_ESM.pdf]
